# Ionic Fragments and Clusters Produced by Electron Impact of Acetonitrile and Methanol Mixed Molecular Films

**DOI:** 10.1021/acs.jpca.4c08285

**Published:** 2025-04-02

**Authors:** Wania Wolff, Andre M. R. Giraldi, Jorge H. C. Basilio, Fabio de A Ribeiro, Alvaro Nunes Oliveira, Ricardo R. Oliveira

**Affiliations:** † Physics Institute, Federal University of Rio de Janeiro, Rio de Janeiro, Rio de Janeiro 21941-909, Brazil; ‡ Federal Institute of Rio de Janeiro, Nilópolis, Rio de Janeiro 26530-060, Brazil; § Chemistry Institute, Federal University of Rio de Janeiro, Rio de Janeiro, Rio de Janeiro 21941-909, Brazil; ∥ Max-Planck-Institut für Kernphysik, Heidelberg DE-69117, Germany

## Abstract

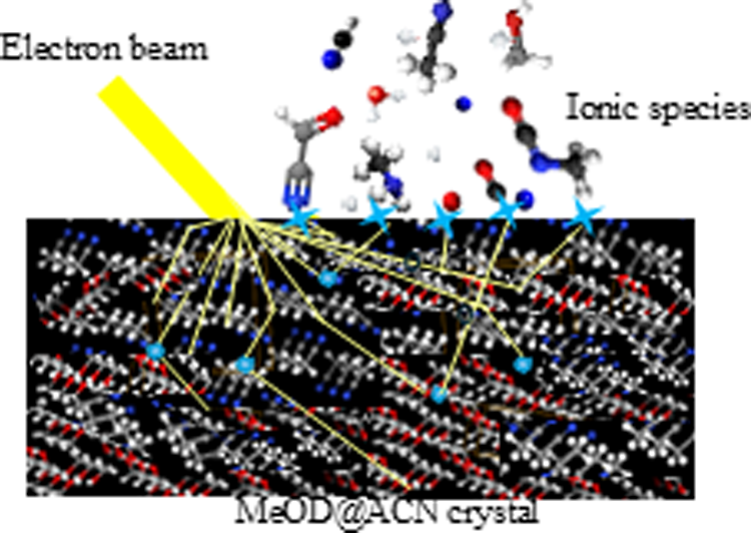

We report the interaction of anhydrous acetonitrile, CH_3_CN (ACN), and deuterated methanol, CD_3_OD (MeOD), in the condensed crystalline phase by electron impact with 2.3 keV of energy. Theoretical and experimental investigations are focused on fragments and aggregates formed as a result of electron-stimulated ion desorption. Positively charged fragments and aggregates were collected using time-of-flight mass spectrometry (TOF-MS) and temperature-programmed desorption based on quadrupole spectroscopy (TPD). The structures of clusters identified in the TOF spectra were studied by applying density functional theory combined with a global minimum search. Two different deposition methods were used for the formation of the condensed molecular films, bilayer and codeposition, and in a second step, the annealing process was performed. The ionic species released from the surface into the vacuum are highly dependent on the annealing. A discussion of the interaction between the molecules was made. The formation of complex organic species comes from the intermolecular or intramolecular interactions of pure MeOD and ACN molecules. Anhydrous compounds were used, and the background water content was minimized to inhibit caging of the ACN molecules by water molecules.

## 1 Introduction

It is well established that chemical reactions triggered by the impact of primary charged particles and photons can generate complex molecular species mediated by secondary electrons released when primary radiation interacts with a dense medium. These electrons efficiently initiate ionization and excitation and lead to molecular fragmentation and the synthesis of complex molecules.^1^

The main goal of the present work was to certify the specificity of the production of ionic species by their sensitivity to film deposition and annealing. Electron-stimulated ion desorption of the surface-produced molecules is responsible for the release of fragments and aggregates into the gas phase. Therefore, the ionization-driven chemistry of freshly sublimated cold COMs can trace molecules and their abundances that were previously in the condensed phase.^2−5^

Models have been developed as a means of describing processes that lead to ionic desorption stimulated by electron impact (ESID).^1,6−8^ The Menzel-Gomer-Redhead (MGR) model based on excitation and quenching processes^1,6^ and the Knotek and Feibelman (KF) model^7,8^ grounded on hole formation, Auger decay, and Coulomb repulsion should be noted.

Nitriles and alcohols are widely used as solvents in research and industrial processes. Methanol, CH_3_OH (MeOH), along with water and carbon monoxide, is among the most abundant molecules identified in the universe. Methanol has been detected in astrophysical objects such as the Taurus Molecular Cloud (TMC-1),^9,10^ circumstellar disk CRBR 2422.8–3423,^11^ outburst V883 Orisystem,^12^ 67P/Cheryumov-Gerasimenko and Hale-Bopp comets,^13−15^ and protoplanetary disk TW Hydrae.? Implications of its presence in gaseous, liquid, and solid phases are discussed on Saturn moons, Titan, and Enceladus, which act as an antifreeze compound in oceans or undergo cryovolcanic activity.^16−22^ Upon the incidence of low- and high-energy electrons on condensed methanol, a variety of more complex molecules have been detected, such as acids, ethers, and larger alcohols.^5,23−27^

Acetonitrile, CH_3_CN (ACN), is present in the molecular cloud TMC-1,^28^ low mass protobinary object IRAS 16293-2422,^28^ in the atmosphere of planetary objects, such as Titan, and in the coma of comets, such as Hale-Bopp^29,30^ and 67P/Churyumov-Gerasimenko.^31^ Recent studies have suggested that condensed ACN subjected to the incidence of electrons and ions leads to the formation of amino acid precursor nitriles, such as methylamine CH_3_ NH_2_ and ethylamine CH_3_ CH_2_ NH_2_.^32,33^

Both molecules (MeOH and ACN) were detected in the same astronomical environments, such as in the molecular dark cloud L1157 outflow,^34^ in cold clouds on the southern part of the core of TMC-1,^28,35^ in Fu Ori outburst of the young star V883 Ori,^12,36^ in comets Hale-Bopp and 67P/Churyumov-Gerasimenko,^14,31,37^ and both may be present in solar planetary moons Titan^21,38^ and Enceladus.

These astrophysical objects are bombarded by low- to high-energy electrons, among solar wind, cosmic-ray, and interstellar radiation sources. Titan and Enceladus moons are exposed to Saturn and Jupiter’s magnetosphere electrons,^16,18−21,39,40^ Hale-Bopp comet to the solar wind and cosmic ray electrons,^41^ and the interior of TMC-1 to secondary electrons.^42^

Based on the astrochemistry data and astrophysical observations here outlined, we present a mass spectroscopic study of condensed ACN and MeOD mixtures by keV electron impact. The ionic fragmentation and aggregation patterns can be held to be indicative of the interaction of these two molecules after electron-molecular collision. Focusing on the molecules of biological relevance, a search was conducted in the astronomical database of organic compounds^43^ detected with similar chemical composition as the presently detected aggregates.

The review is organized as follows. Briefly, we describe the experimental and theoretical methods. Then, to get a better understanding of the desorption process of the condensed layers, we performed TPD measurements of the codeposited and bilayer mixtures. In the next section, we present the experimental results based on mass spectrometry of the ionic species formed from mixtures of acetonitrile and methanol. Two different deposition procedures were applied to probe their interactions in the solid state. The mixtures were also subjected to an annealing process to promote the mobility of both molecules in the crystalline structure. The yields of production of ionic species from the mixtures were compared to those of the pure samples to identify complex species with confidence. Finally, calculations of the global minimum (GM) of ionized states are presented and compared to those of molecules detected in astrophysical environments.

## 2 Experimental Details

The setup consists of an ultrahigh vacuum stainless steel chamber with different instruments attached to its ports. The electrons were delivered by a commercial electron gun (ELG-2—Kimball Physics), and the ionic species were collected by a homemade linear time-of-flight mass spectrometer and a residual gas analyzer (RGA-SRS-200—Stanford Research System). At the top of the chamber, an XYZ sample manipulator provided precise positioning of the stainless steel sample holder. It was cooled by an open-cycle liquid nitrogen cryostat to temperatures around 120 K. The substrate temperature was controlled and monitored by a K-Type thermocouple connected to an Eurotherm 2408 sensor controller. The chamber connected to a 350 L s^–1^ turbo pump was maintained at a base pressure of 1.0 × 10^–9^ mbar reached by titanium sublimation pumping. The hot and cold cathode pressure sensors measured the pressure in the chamber. The injection line connected to the chamber was kept around 1.0 × 10^–3^ mbar, heated, and dried by nitrogen gas flux prior to injection to avoid water contamination. Water is known to cage ACN molecules in a clathrate-like structure, preventing them from interacting with the other molecules in the poly crystalline structure.^44^ Furthermore, water layers can also alter the structure and desorption pattern of pure molecules.^44^

Acetonitrile and methanol are liquid at room temperature, and their evaporation temperatures at 1 atm are 228 and 176 K, respectively.^45^ Before admission of the evaporated molecules into the chamber, three freeze-pump-thaw cycles were repeated until no bubbles were observed to ensure that all of the dissolved gas or any gas trapped within the frozen solid were removed. Previously, the liquid samples were left in contact with 3A and 4A molecular sieves to be dehydrated, removing water as much as possible. The liquid samples were taken in glass to metal-sealed vials and were connected to separate sample lines. The injection line was flushed several times with vapors of the desired molecules prior to deposition.

The condensed films were grown in situ by the admission of the evaporated molecules into the chamber through two different precision all-metal leak valves. The films were deposited from a diffuse background flux, and thus the prepared film was porous.^46^ The base pressure was increased in the range of 10^–8^ mbar for 10 min after admission, and the vapor injection process was monitored by a QMS-RGA set at 70 eV electron energy. The liquid evaporation rate in the injection line was monitored by Pirani pressure sensors and remained constant for the time of the injection. All control and monitoring procedures were based on the LabVIEW platform. The intensities of the relevant mass species (H_2_O, N_2_, and O_2_), the parent ion, and the more intense fragments of ACN and MeOD were collected and registered in pressure versus time by the RGA (spectra available in the Supporting Information). Total ion production was estimated considering the absolute cross-section values of MeOD, ACN, and H_2_.^47−50^ The sticking factor for ACN and MeOD listed in the literature is unit.^51,52^ The pumping speeds of ACN and MeOD can be considered similar for the turbo pump used and relative small mass differences.^53^ We considerably reduced the presence of light gases (mainly H_2_ and water) in the system by using a sublimation pump.

Two deposition procedures of the molecular films were employed. In bilayer deposition, binary films of ACN@MeOD with acetonitrile (23 L) on top of methanol (9.5 L) were formed by first admitting methanol and then acetonitrile into the chamber by the leak valves. In codeposition, the compounds were admitted simultaneously by the leak valves. Methanol and acetonitrile molecules were deposited on the surface at 16 and 45 L, respectively. One Langmuir (1 L = 1 × 10^–6^ Torr sec) leads to a coverage of approximately one monolayer (1 ML of film thickness) of adsorbed gas molecules on the surface.^54^ For surface coverage greater than 50 ML, the condensed systems should be substrate independent, and the properties are likely to depend only on the molecular environment.^55^ In both cases, after tests according to different injection exposure rates, the optimal value used in the experiments presented was 1:3 (MeOD/ACN). This ratio, among the tested ratios, was the most responsible for the activation of channels of intermolecular interaction and, thus, ionic species formation. The condensed films were fast warmed by resistive heating of the sample holder to 135 K to enhance the mobility of the molecules in the film and the intermolecular interaction. The threshold temperature was established by the TPD process to avoid sublimation of the multilayer component of the film. The thickness of each layer can be roughly estimated by assuming that a monolayer has dimensions very close to the dimensions of the unit cell of each molecular crystal. For methanol, the lattice constants *a* = 4.71 Å, *b* = 4.93 Å, and *c* = 9.13 Å have been determined by He et al.^56^ for ultrathin films using electron diffraction. Considering that molecular layers are vertically stacked along the *c* direction, it is possible to estimate the total thickness of the MeOD layer deposited in this work as ≈ 8.7 nm. Likewise, the lattice constants of the ACN single crystal have been derived for the α phase by^57^ as *a* = 4.102 Å, *b* = 8.244 Å, and *c* = 7.970 Å. Adopting the *c* lattice constant as the direction of stacking and from the exposure employed here at 120 K, the thickness of the ACN layer is estimated to be ≈ 18.1 nm over the previously deposited MeOD layer, resulting in an upper limit of ≈ 26.8 nm estimated for the total thickness of the layered ice.

The films were impacted by a pulsed electron beam with a final energy of 2300 eV, with a pulse width of 20 ns within a time repetition rate of 80 kHz (12.5 μs). A time-averaged current of 2 nA delivered 1.1 × 10^5^ incident electrons. The beam current was measured at start and stop of the experiments and was shown to be very stable. The total number of pulses (sweeps that triggered a time-to-digital converter, TDC-Multistop, FAST ComTec Gmbh) was usually set to ∼10^8^. The real time of the experiments was ∼20 min. The experimental conditions allowed probe of desorption with a relatively low fluence of 0.3 × 10^11^ electrons cm^–2^ to mitigate surface decomposition.^44^ The ion count rate on the electron multiplier detector was monitored and maintained at 4–5 kHz.

Positively charged fragments, which desorb from the sample surface upon electron impact, were measured by time-of-flight mass spectrometry (TOF-MS). The charged ions were extracted from the interaction surface by applying a positive potential (+1900 V) to the sample holder, which defined the electron impact energy of 2.3 keV (the electron gun output energy was +400 eV). The ions were focused by a lens set at +1350 V, traveled through the field-free-flight tube of 25 cm, and collected by an MCP detector in Chevron configuration. The signals were processed by NIM fast standard electronic modules and directed to a fast multistop P7886 TDC (1 ns). The TDC was triggered by the start input signal given by the electron gun pulser. The sweep ended with the arrival of the ion signal, which gives the stop input signal or the end-of-sweep dead-time signal. Each event is recorded by MCDWin software that generates the time-of-flight spectra.

The gun exit was located at a distance of 5 cm from the holder (substrate). The sample holder was placed perpendicular to the spectrometer 1 cm from its entrance. The focused electron beam hit the sample at an incidence angle of 60° to the normal over an area of 0.5 mm^2^. The electron beam profile and beam spot at the holder were visualized with a sensitive electron cesium crystal. The position and angles of the holder with respect to the spectrometer and the profile of the beam greatly affected the spectral resolution. Therefore, the settings were optimized with the help of the TOF spectra.

Measurements were repeated in slightly different positions on the film. Possible sublimation of the molecular film was tested using 70 eV electrons delivered by a quadrupole mass spectrometer (QMS) RGA. None of the sublimated ions of the condensed film molecules were detected before and after the incidence of the electrons. The sublimation rate of ACN and MeOD was considered negligible at the 10^–9^ mbar pressure level.

Temperature-programmed desorption (TPD) was started by recording the evolution of selected ionic species at a temperature ramp rate of 0.1 K/s. The film layers were removed by resistive heating and monitored by the QMS-RGA. The TPD procedure was used to monitor the evaporation of the molecules from the substrate surface before a fresh film was deposited under conditions similar to those of the previously optimized one.

Electron trajectory simulations made with the CASINO code revealed that 2.3 keV electrons can penetrate to a maximum depth of up to 66 nm within the layered sample, and the maximum penetration depth distribution occurs very close to the interface between MeOD and the sample holder (substrate) at 31.7 nm. Given that the thicknesses of the deposited films were estimated to be smaller than these values, it is feasible to conclude that the majority of the electrons can transverse the film and be dumped in the substrate. Detailed information on the simulations can be found in the Supporting Information.

It is noteworthy that although incident electrons can excite and ionize molecules in the underlying layers close to the metallic substrate, it is quite unlikely that ions created very deep inside the molecular film can make it to the surface before being neutralized. Thus, only those ions that are emitted very close to the surface–vacuum interface can be detected by a time-of-flight mass spectrometer. This makes the detection of ions and ionic clusters a signature of the physicochemical effects manifested on the surface of the film following the incidence of electrons.

Fully deuterated methanol CD_3_OD (MeOD—*m* = 36 au) and acetonitrile CH_3_CN (can—*m* = 41 au) were deposited on the stainless steel substrate. Deuterated methanol was selected as a means to help identify which molecule provided which radicals to the clusters. In both mixtures, proton and deuterium transfer can proceed from acetonitrile to methanol, in an opposite way or even within the same respective components, and structural preferences take place. There are two possible sources of D in methanol molecules, the OD or CD_3_ group, leaving either CD_3_O or CD_2_OD radicals behind, while in acetonitrile, proton transfer can only proceed through the CH_3_ group. Therefore, the deuteration allows us to discuss the major spectral changes and provide a picture of the processes induced by electron impact ionization.

## 3 Computational Details

The search for the global minimum (GM) structures of molecular clusters produced by the interacting samples was performed via a two-step process. The initial structures were generated and optimized using a combination of two softwares: ABCluster and xTB. The ABCluster software^58^ is based on a swarm optimization algorithm, which simulates the foraging behavior of bees, that is, their behavior in the search for the best food sources (in this case, low energy geometries). xTB^59^ is a software in which the extended tight-binding method is implemented. This method is parametrized to reproduce accurate results with respect to molecular geometries, vibrational frequencies, and noncovalent interactions. Due to the relatively accurate results and computational efficiency, the energy and gradient calculations performed by xTB were combined with the ABCluster algorithm.

The first step of the GM search and structure optimization is dependent on the molecular complexity and stoichiometry. At least 1000 geometry optimizations were performed for each molecular structure. The first 100 low-energy isomers for each analyzed cluster were further reoptimized in the second step of the GM search by applying the ORCA software at the density functional theory (DFT) level. The ORCA software^60^ was used for optimizations of structures and vibrational frequency calculations applying the PBE0 functional^61^ combined with the def2-TZVP basis set. The PBE0 is considered an accurate model with respect to molecular and spectroscopic properties, such as molecular geometries and relative energies.^62−65^ After this second step of the GM search, all calculated structures were organized in the ascending relative energy order, considering the sum of the electronic and zero-point energies (ZPE). Moreover, low-energy isomers of each cluster are presented and discussed in the “Global minimum structures” subsection. It is important to note that all optimized structures are in the cationic charge state (*q* = +1) and have the lowest possible spin multiplicities.

## 4 Results and Discussion

All experiments were started under similar conditions: sample temperature (120 K), beam current (2 nA), and ion count rate (4 kHz). The results of temperature-programmed desorption (TPD) analysis and electron-stimulated ion desorption (ESID) of the condensed deuterated methanol—acetonitrile mixtures are shown first.

The analysis of ionic production was performed by comparing the yields of production of ionic species between the pure samples and the mixtures, highlighting the differences in the mixtures due to the annealing process. The yields of the ions were extracted by integrating the corresponding TOF peaks (the mass-to-charge ratio spectra are available in the Supporting Information). The comparison between the pure films and each deposited mixture indicates whether the bilayer or the codeposition was responsible for a greater exchange of H and D atoms and a greater intermolecular interaction between the compounds. The greater the interaction, the greater the production of the so-called “mixed clusters”. These clusters are assigned to species formed by a methanol molecule associated with an acetonitrile fragment or vice versa. The ions, which resulted from the mixed MeOD/ACN compounds, were used as an aid to probe the intermolecular interactions of MeOD and ACN.

The computational results that will be presented concern the optimization of molecular structures and the search of global minima of possible MeOD/ACN clusters identified in the mass spectra. The resulting structures were compared with molecules that have already been detected in interstellar environments.

### 4.1 TPD

TPD measurements of molecular films can indicate the amounts of MeOD and ACN deposited on the holder and can help explain the interaction between molecules in the mixtures driven by the initial electrons. MeOD and ACN are identified in the TPD spectra corresponding to the molecular mass of the ionized molecules (*m*_MeOD_ = 36 au and *m*_ACN_ = 41 au). The intensity of the desorption peak is proportional to that of the neutral precursor molecule on the surface. TPD was also used to define the threshold temperature for diffusing the molecules in the film to promote molecular rearrangement on the surface and in the bulk of the condensed films. This information is essential to avoid sublimation of the samples during the annealing procedure.

At a temperature of deposition of 120 K, both molecules have crystalline structures, since their amorphous phase is only reached at temperatures around 100 K.^39,66−69^ Methanol is known to possess two crystalline phases, α and β, with a transition temperature between 155 and 165 K with increasing temperature.^69,70^ Studies have suggested the existence of a metastable phase in the temperature range between the α and amorphous phases,^69,71^ while others found evidence that this metastable structure might be a mixture of crystalline phases α and β.^72^ Taking into account the present deposition procedure, the deposited methanol film must be in the α-phase form.^39,69,70^

Acetonitrile also has two distinct crystalline phases: a high temperature (HT) phase and a low temperature (LT) phase, with a transition temperature between both structures around 217 K.^67,68,73−76^ The HT morphology has antiparallel ACN dimers as the basic unit, while in the LT phase, the molecules of the dimer are perpendicular to each other’s molecular axis. The deposition of ACN on substrates with temperatures around 120 K gave form to a LT-structured film.^67^ Therefore, the ACN films deposited in this study can have an LT morphology.

It is clear that the deposition temperature, composition of the substrate (and roughness), and deposition rate affect the morphology of the film. However, further investigation must be performed before conclusive identification of the structure of the film can be established.

**Figure 1:**
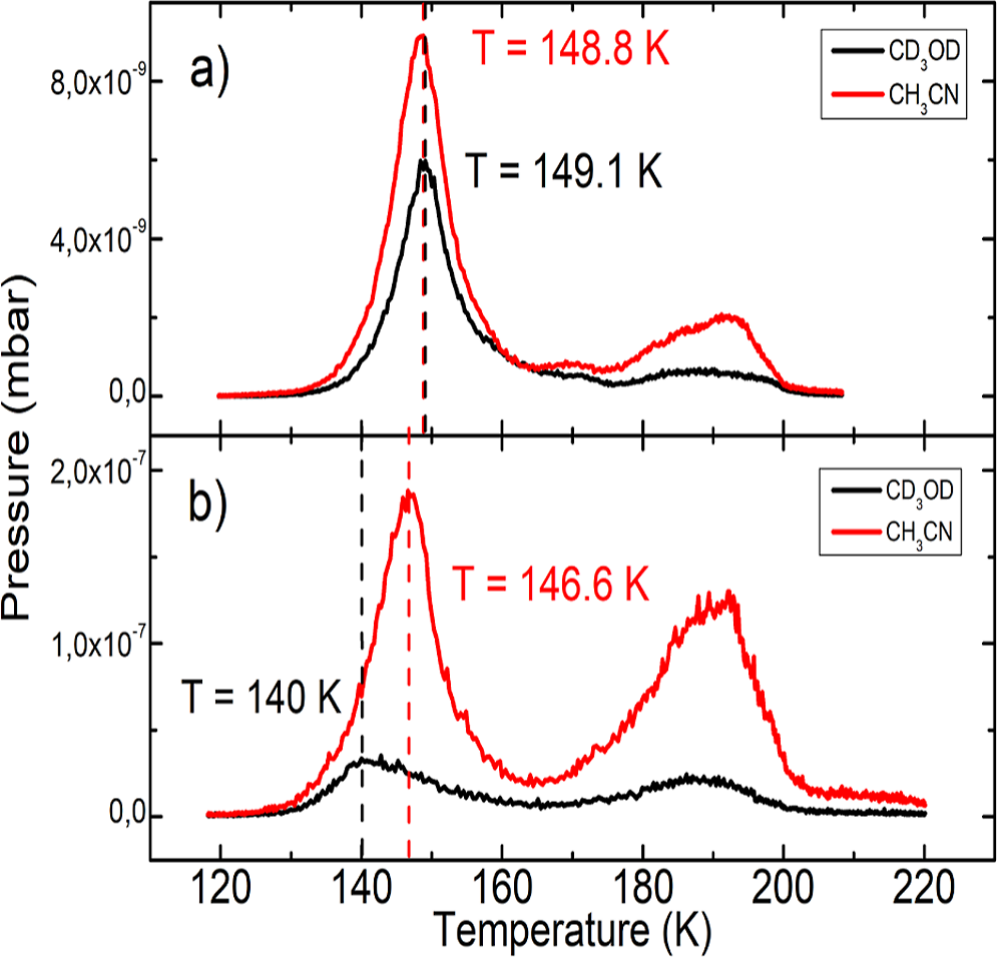
TPD of the (a) codeposited and (b) bilayer ACN and MeOD mixtures. The red and black lines show, respectively, the evolution of the *m*/*z* = 41 and *m*/*z* = 36 partial pressures (mbar) as a function of the temperature (K).

The desorption structures present in the TPD spectra in Figure 1 are attributed to the physisorbed layer of the films.^44,55^ The first desorption peak present in the TPD spectra is due to thermal desorption of methanol α and the LT phase of acetonitrile. A comparison of the thermal desorption pattern between the pure films (Figure S2a,b) and the codeposited and bilayer films (Figure 1) shows an increase in the temperature of the first desorption peak on both mixtures. In the codeposited mixture, the desorption temperatures of acetonitrile and methanol were quite similar ∼149 K, with similar shapes and intensities. However, in the bilayer film, the MeOD signal peaks at 140 K, while the ACN only peaks at 147 K, and the intensities of the signals are quite different. The desorption temperature shifts are consistent with the data from previous work.^55^ The results suggest a stronger intermolecular interaction between ACN and MeOD within the codeposited mixture and can justify the enhanced emission of mixed-ion clusters observed in the TOF-MS spectra. The pure films and mixtures show a second high peak. These high-temperature signals probably stem from molecules or fragments in immediate contact with the metal holder. The detailed investigation of this effect was beyond the scope of the present work and, therefore, was not carried out.

### 4.2 Deuterated Methanol—Acetonitrile Mixture

**Figure 2:**
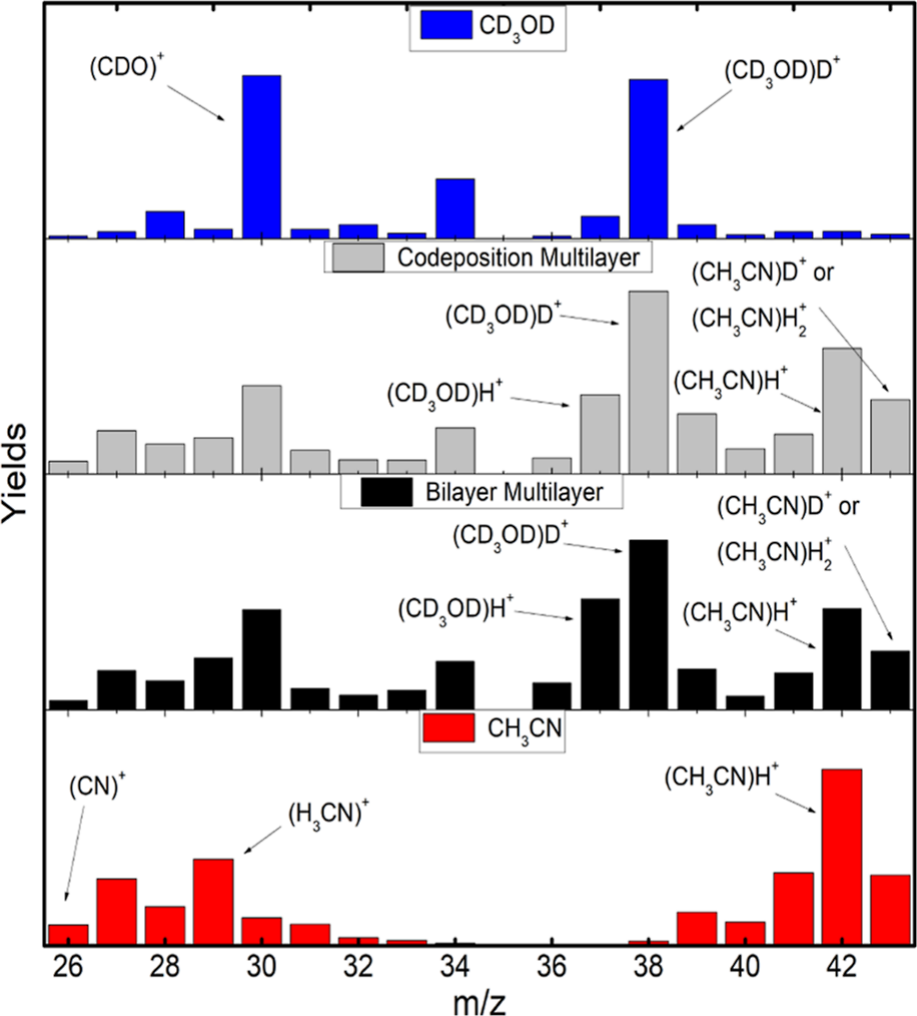
Yield comparison, from top to bottom, of pure MeOD, codeposited mixture, bilayer mixture, and pure ACN in the range of *m*/*z* from 26 to 43.

**Figure 3:**
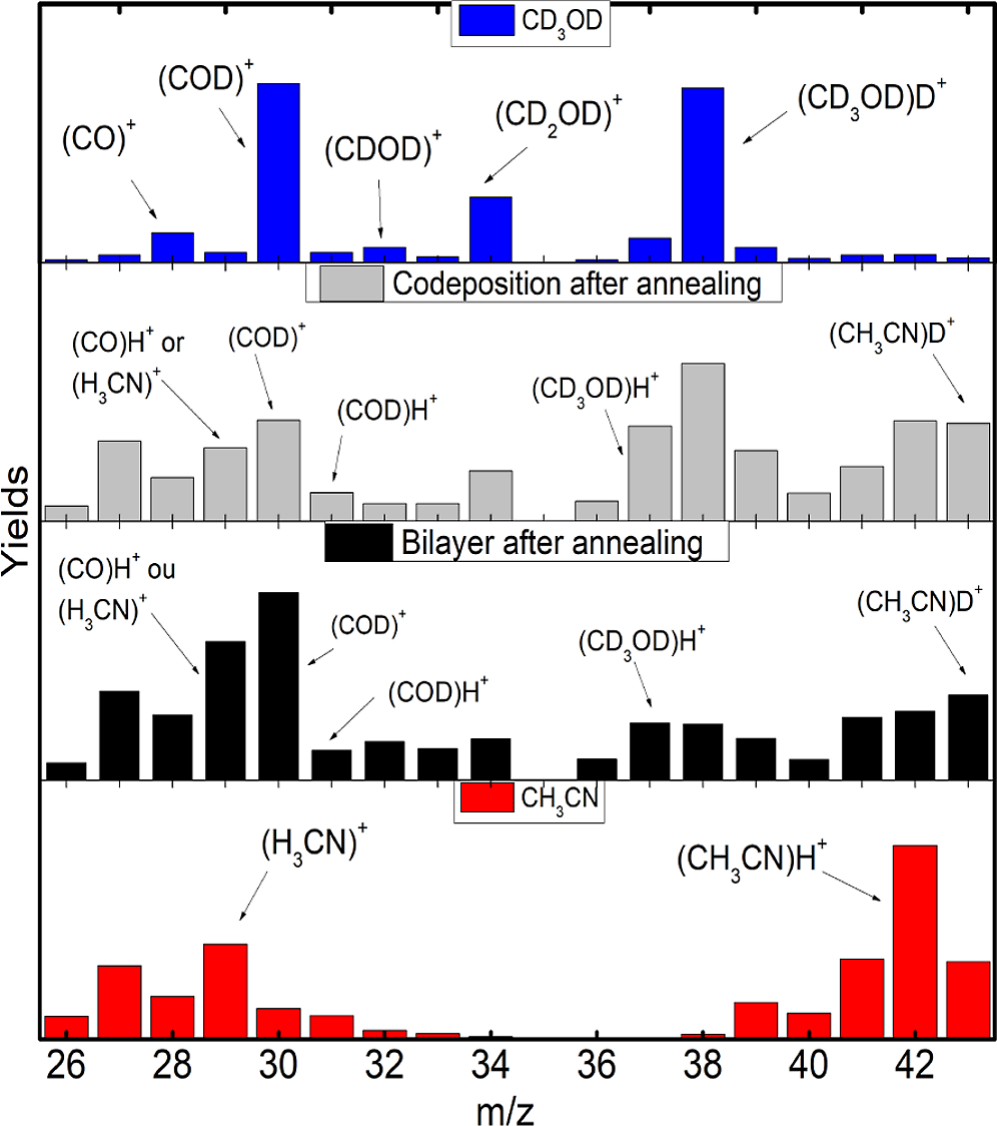
Yield comparison, from top to bottom, of pure MeOD, codeposited mixture after annealing, bilayer mixture after annealing, and pure ACN in the range of *m*/*z* from 26 to 43.

**Figure 4:**
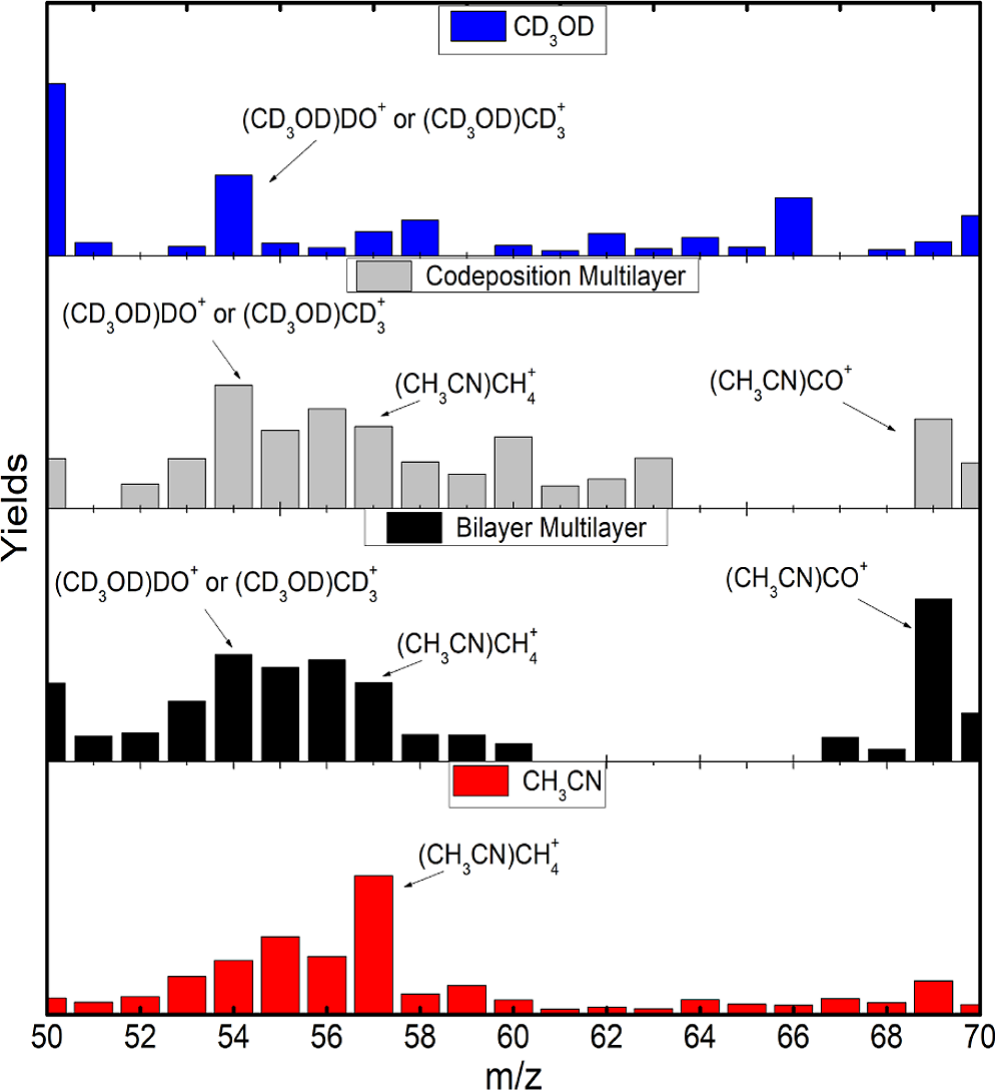
Yield comparison, from top to bottom, of pure MeOD, codeposited mixture, bilayer mixture, and pure ACN in the range of *m*/*z* from 50 to 70.

**Figure 5:**
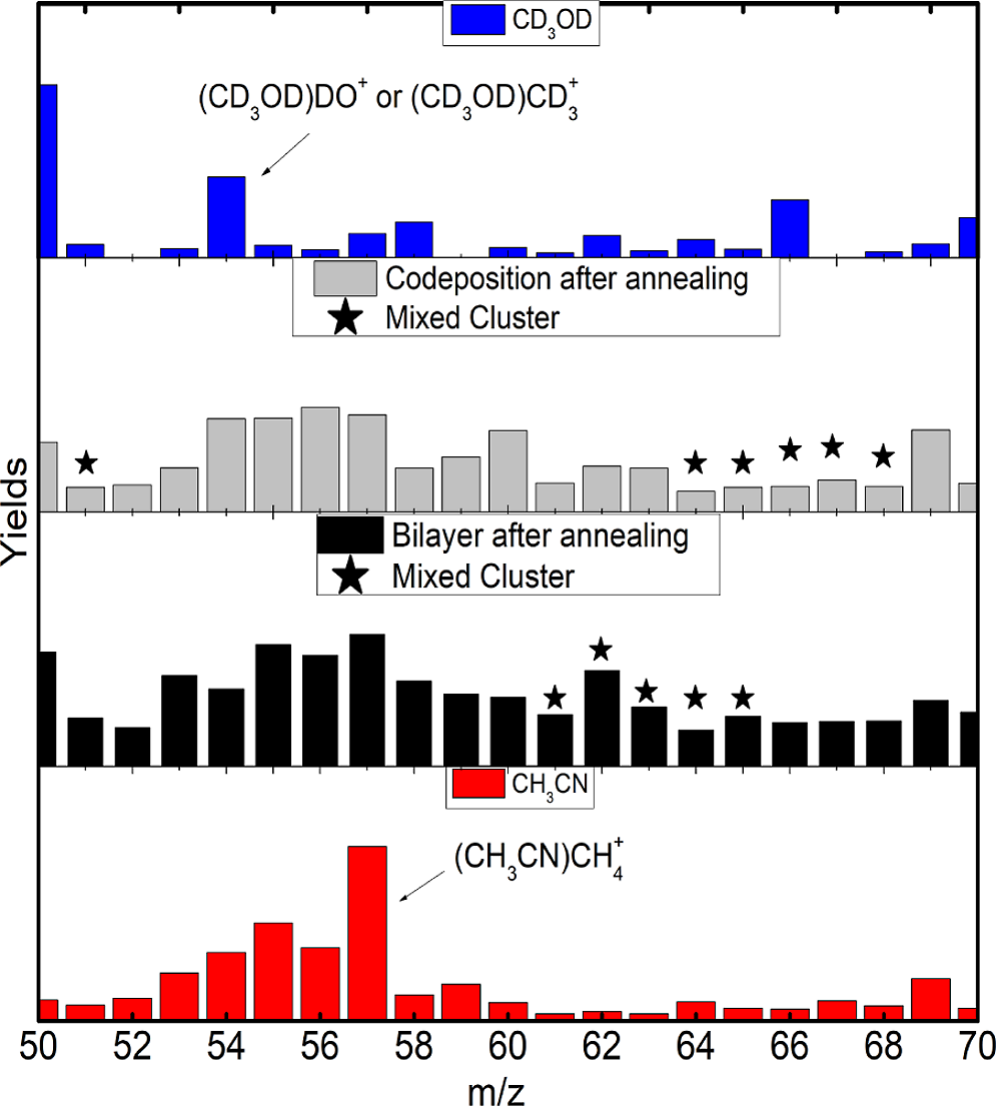
Yield comparison, from top to bottom, of pure MeOD, codeposited mixture after annealing, bilayer mixture after annealing, and pure ACN in the range of *m*/*z* 50 to 70.

**Figure 6:**
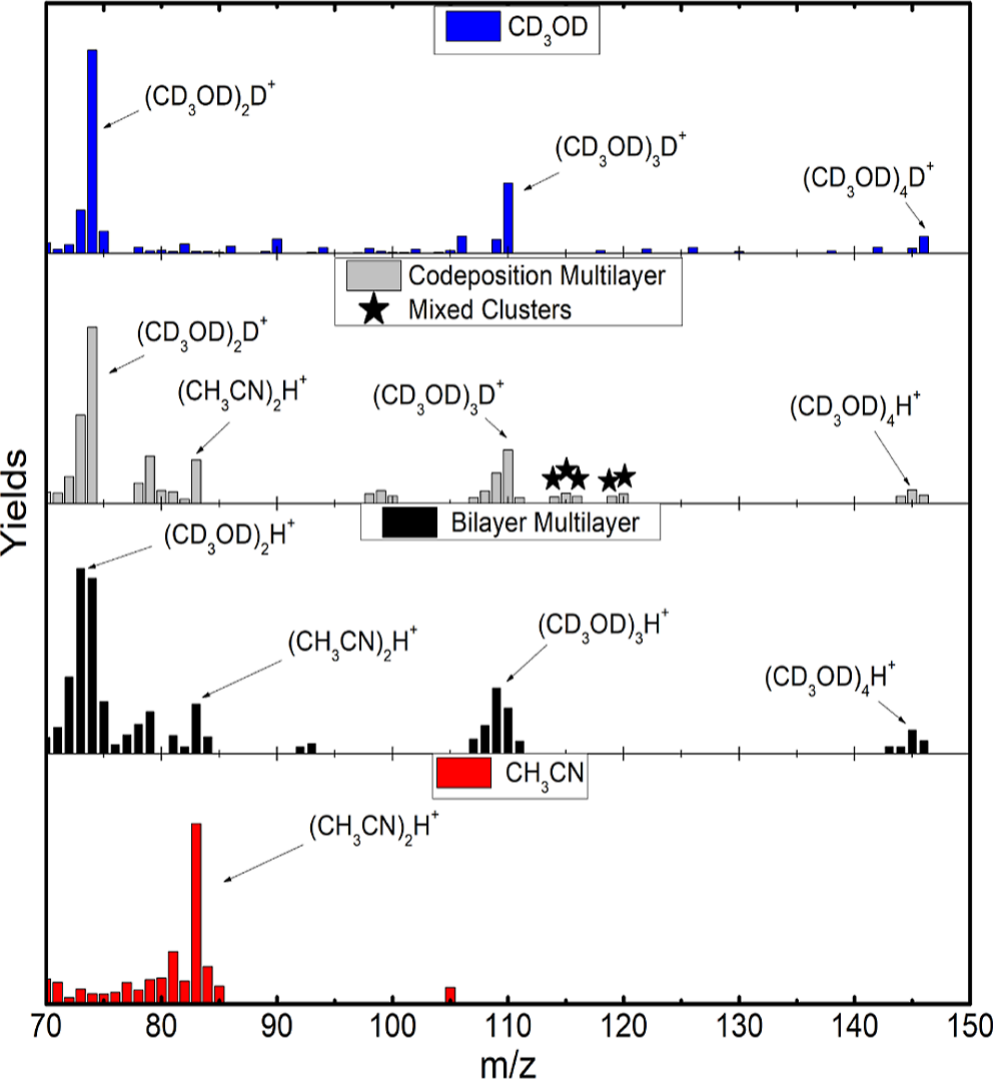
Yield comparison, from top to bottom, of pure MeOD, codeposited mixture, bilayer mixture, and pure ACN in the range of *m*/*z* 70 to 150.

**Figure 7:**
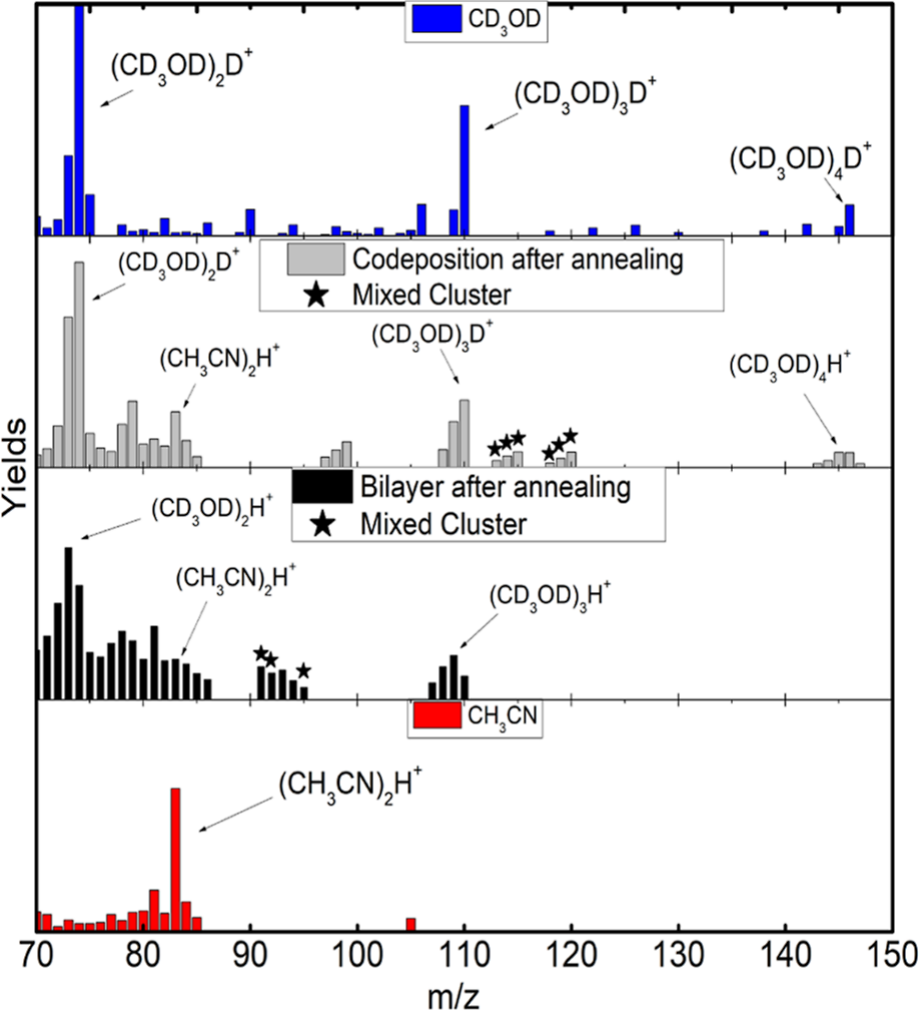
Yield comparison, from top to bottom, of pure MeOD, codeposited mixture after annealing, bilayer mixture after annealing, and pure ACN in the range of *m*/*z* from 70 to 150.

Care was taken to maintain similar experimental conditions for the measurements of the mixtures. However, it is not feasible to ascertain that absolute equal conditions were present at the film deposition, and some deviations were unavoidable in the experiments. Therefore, the yield scales are omitted in Figures 2, 3, 4, 5, 6, and 7. The comparison of yields between the multilayer and annealed films of the same mixture is meaningful, as is the pattern evolution (growth or decay) of the same species. The yields of the pure samples are included in the comparative analysis, indicating the production of distinct species in the mixtures.

The comparison was divided into different ranges of the mass-to-charge ratio (*m*/*z*) for pure MeOD, pure ACN, and before and after the annealing process on the MeOD/ACN mixtures. The first range includes the ionic fragments of *m*/*z* = 26 and the hydrogenated species of the ionized molecules. These yields before the annealing process are presented in Figure 2. The comparative analysis intends to point out the yields of the masses of marked relevance.

Deuterated methanol produces mostly even mass species, but still some ions with odd masses are present because of some D/H exchange in the chamber. Ionized methanol (MeOD^+^) has a small yield, due to its tendency to attach deuterium forming (CD_3_OD)D^+^. Deuterium ions (D^+^) can easily overcome the surface potential of the condensed film. As hydrogen ions are less available on the surface, the yield of protonated species (CD_3_OD)H^+^ is smaller. A high yield of CDO^+^ (*m*/*z* = 30) is derived due to the formation of a closed-shell species. Yields ratios are present in Table S1 for a better comparison between (CD_3_OD)_*n*_H^+^ and (CD_3_OD)_*n*_D^+^ formation.

Pure acetonitrile can form even and odd-mass ions. The species in the range of *m*/*z* = 26–34 can be related mainly to the production of C_2_H_*n*_^+^ and CNH_*n*_^+^. However, the strong alternation between even and odd-mass ions indicates a higher contribution of the first series. Closed shell ions with an odd number of hydrogen atoms are more stable than species with an even number of hydrogen atoms in that series.^77−79^ The protonated acetonitrile (CH_3_CN)H^+^ is preferentially produced, while the ionized ACN (CH_3_CN^+^) and ACN bonded to two hydrogen atoms, (CH_3_CN)H_2_^+^, are less produced, showing similar yields.

The fragment range with *m*/*z* = 26–43 showed similarities between the codeposited and bilayer spectra before the annealing process. The MeOD and ACN molecules preferentially attach themselves to hydrogen or deuterium, and therefore, an increase in the yields is expected. This clearly indicates an interaction between both molecules since the hydrogen comes mainly from acetonitrile, and the deuterium comes only from the MeOD. The interaction between them is due to the hydrogen bonding between the deuterated hydroxyl group (OD) of methanol and the cyanide group (CN) of acetonitrile.^55^ Another work suggested that the interaction could be induced doubly, one between a hydrogen from ACN’s methyl group and the oxygen atom from methanol, and the other between ACN’s nitrogen atom and a deuterium from the methyl group of methanol.^80^ This double interaction could explain the exchange of H and D between the molecules.

In both mixtures, the presence of odd mass ions in the range of *m*/*z* = 26–34 can be attributed to the ACN molecular fragments and to the hydrogenation of the MeOD fragments. Because the methanol fragments are due to the loss of deuterium, which preserves the C–O bond, hydrogenation comes mainly from an interaction between the oxygen and a hydrogen from the ACN’s methyl group.

After the annealing process, the production of deuterated ACN and protonated MeOD is promoted in both mixtures (Figure 3) and reflects the interaction between the two molecular compounds. The intermolecular association competes with the intramolecular one, and they determine the reduction of the protonation of ACN and the deuteration of MeOD. In the bilayer film, the yields of these both protonated and deuterated ACN and MeOD species are very similar and indicate a greater exchange of hydrogen and deuterium atoms between the molecules than before the annealing.

Compared with before the annealing, the codeposited film shows an increase in the yields of odd-mass fragment ions, particularly those related to MeOD fragments associated with hydrogen atoms. In the bilayer film, a reduction in the yields of protonated MeOD and ACN and deuterated MeOD is observed after annealing. This decay in the yields may explain the high yields in the fragment region caused by the breakup of the molecules.

The second range covers the yields of ionic aggregates with *m*/*z* = 50–70 and is shown in Figure 4. High-mass radicals are attached to CD_3_OD and CH_3_CN. Pure MeOD shows the highest cluster yields when attached to C atoms, O atoms, and radicals such as CD, OD, CD_3_, CO, CDO, CD_2_O, and CD_3_O (*m*/*z* = 50, 54, 58, 62, 66, and 70). Pure ACN, on the other hand, has as major yields an attachment to C atoms and hydrogenated radicals of C, such as CH_2_, CH_3_, and the CH_4_ molecule, which form the series (CH_3_CN)CH_*n*_^+^ (*n* = 0–4). The cluster (CH_3_CN)H_2_CN^+^ of *m*/*z* = 69 is also produced and is related to the incorporation of hydrogen cyanide (HCN).

In the multilayer regime, that is, before annealing, the high yields on the mixtures correspond to the sum of aggregates of pure MeOD and ACN. The production of mixed species is not the rule, indicating that MeOD and ACN seem to be segregated in the film. From the yield analysis shown in Figure 4, some species are even absent in the mixtures, although they were formed in the pure films. In the codeposition, aggregates of *m*/*z* = 64–68 were absent, while in the bilayer film, species with *m*/*z* = 61–66 were not present. The only species of mixed cluster of relevance is (CH_3_CN)CO^+^ (*m*/*z* = 69).

After annealing, the yields of the mixtures presented in Figure 5 show the continuous formation of ionic species in the range of *m*/*z* = 50–70. MeOD associated with a fragment of the ACN and vice versa produces mixed clusters, which are identified in the spectra by black stars. The annealing process breaks down the segregation of the molecules, inducing the interaction between them. Examples of possible assignments are (CH_3_CN)D_2_O^+^ (*m*/*z* = 61), (CD_3_OD)CN^+^ (*m*/*z* = 62), and (CD_3_OD)HCN^+^ (*m*/*z* = 63). The search of GM structures for some of these species was carried out to identify possible molecular formation. The results are discussed in the “Global Minimum Structures” subsection.

Figure 6 shows the yields of larger and more complex clusters with *m*/*z* = 70–150 before annealing. Methanol is known for its clusterization,^81^ as evidenced by the high yields of the dimer, trimer, and tetramer attached to deuterium (see Table S1). In contrast, only the protonated ACN dimer (CH_3_CN)_2_H^+^ is identified in the pure ACN spectrum.

In the codeposition, the MeOD clusters attach more hydrogen atoms of ACN. The production of the hydrogenated tetramer (CD_3_OD)_4_H^+^ is able to overcome that of the deuterated one. This conversion becomes more pronounced in the bilayer film, where the yields of methanol’s aggregates (CD_3_OD)_*n*_H^+^ stand out (see Table S1). In both mixtures, the protonated ACN dimer is present.

In terms of the production of mixed clusters prior to annealing, aggregates identified as such in the codeposited film were not formed in either of the pure spectra. They may indicate species produced by the association of multiple ACN and MeOD molecules. Possible assignments are (CD_3_OD)_2_(CH_3_CN)^+^ (*m*/*z* = 113) and (CD_3_OD)(CH_3_CN)_2_^+^ (*m*/*z* = 119). No mixed species were observed in the bilayer film, suggesting that the species come from the interaction of the two layers of isolated MeOD and ACN.

In both mixtures, annealing increased the production of mixed clusters (Figure 7). Subsequent peaks after the protonated ACN dimer may arise from the attachment of more H/D atoms. On the other hand, the methanol tetramer is not produced in the bilayer mixture, supporting the greater breakup of MeOD molecules in this regime.

An explicit attribution to mass-to-charge ratios to possible molecular structures is provided in Tables S2–S4.

### 4.3 Global Minimum Structures

**Figure 8:**
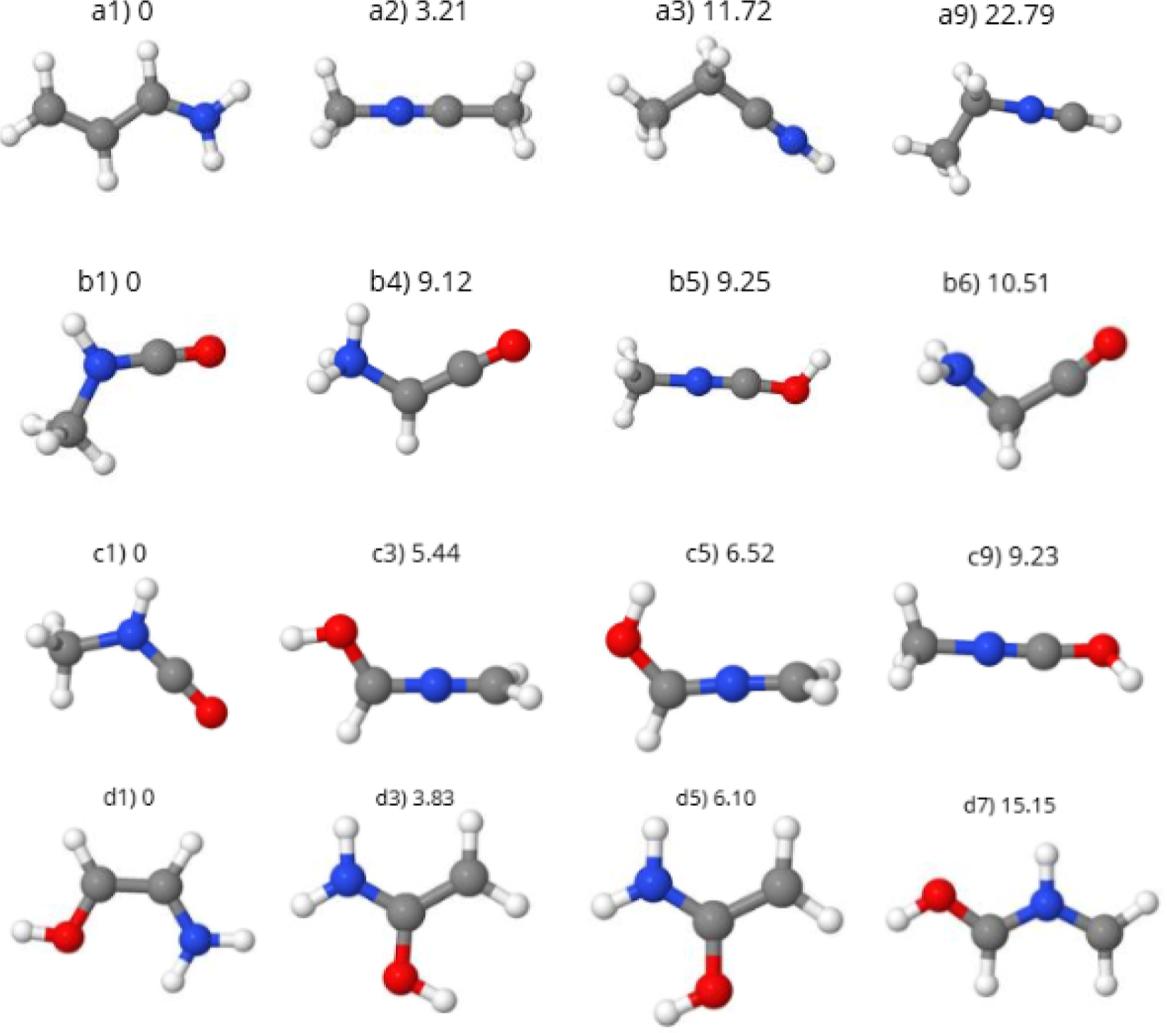
Calculated GM structures as products from several reactions. The numbers above each structures are the relative energies in kcal mol^–1^. Carbon atoms are gray, hydrogen atoms are white, nitrogen atoms are blue, and oxygen atoms are red. Structures from the first to the last (forth) line are associated with *m*/*z* = 59, 59, 62, and 63 fragments, respectively.

The global minimum (GM) structures of each of the selected molecular clusters are listed in Figure 8. The optimized clusters are associated with one of the molecules bonded to a molecular fragment from the other compound and are here defined as “mixed clusters”. The 10 low-energy isomers for each case were calculated, and some are presented in the ascending relative energy order (for a complete list of the structures considered, see the Supporting Information from Figures S5 to S8). They are compared with species identified in astronomical environments according to the McGuire census.^43^ The calculations did not distinguish hydrogen and deuterium atoms, although deuteration was important in distinguishing each sample’s contribution in the mass spectra and yields. As mentioned above, all geometries have a total charge of *q* = +1.

The reactions occurring upon electron impact in the energy range of keV on the condensed films are far from equilibrium. However, in the present analysis, a thermodynamic equilibrium is assumed.

#### 4.3.1 CH_3_CN + CD_3_ (*m*/*z* = 59)

The cluster formed by a methyl radical from the dissociation of methanol associated with the acetonitrile molecule may be related to the formation of larger nitriles. Structures **a3** and **a9** (Figures S5 and 8) are very similar to the propionitrile molecule CH_3_ CH_2_CN, detected in the ISM,^43^ bonded to one more hydrogen atom. Other structures such as **a1** have a carbon chain that resembles the acrylonitrile molecule CH_2_CHCN, also detected in the ISM,^43^ with hydrogen atoms bound to the CN group.

The same stoichiometry was previously investigated^62^ at the DFT level of theory. Regarding the global minimum structure, they found a similar geometry of **a2** (this work) with the methyl radical attached to the nitrogen of the CN group. This result corroborates the suggestion that the intermolecular interaction between ACN and MeOD is induced doubly^80^ by the nitrogen atom of ACN interacting with one of the hydrogen atoms of the methyl group of methanol. The destruction of the methanol molecule could lead to the desorption of the (CH_3_CN)CD_3_^+^ aggregate. In a previous study, the possibility of intramolecular rearrangement leading to other minima was also mentioned.

#### 4.3.2 CH_3_CN + OD (*m*/*z* = 59)

The structures **b8** and **b9** shown in Figure S6 are the closest to the geometry of acetonitrile, with OH bonded to the methyl group of ACN after hydrogen rearrangement. This process forms a molecule similar to the glycolonitrile (HOCH_2_CN), found in the ISM^43^ and in the molecular cloud G+0.693.^82^ It is interesting to note that none of the low-energy molecular structures represent the ACN molecule with the OD bonded to the CN group.

This cluster has a stoichiometry identical to the methyl isocyanate, CH_3_NCO, detected in ISM,^43^ with one more hydrogen atom. Structures **b1** and **b5** (Figures 8 and S6) represent such species, with hydrogen bonds to nitrogen and oxygen atoms, respectively.

The structure of **b6** is identical to a glycine amino acid precursor (NH_2_ CH_2_COOH). This amino acid has already been detected in comets, such as the 67P/Churyumov-Gerasimenko and Wild 2.^83−85^ Possible reaction pathways for the formation of this amino acid have as a starting point the methylamine (NH_2_ CH_3_) or its radical (NH_2_ CH_2_) (both species produced by acetonitrile irradiation) and carbon monoxide CO (produced by methanol fragmentation).^86,87^ Some of the reaction pathways studied are^88^

NH_2_ CH_2_ + CO → NH_2_ CH_2_CO and NH_2_ CH_2_CO + OH → NH_2_ CH_2_COOH, which also involve the OH group, another product from methanol dissociation. This explains why mixtures of methanol and acetonitrile provide a fertile environment for the formation of glycine amino acid.

The intermediary molecule produced after the first reaction (NH_2_ CH_2_CO) is the same as **b6** and is very similar to **b4**, with a hydrogen atom rearrangement. This indicates the possibility of the formation of glycine from the mixtures of acetonitrile and methanol from the condensed phase.

#### 4.3.3 CD_3_OD + CN (*m*/*z* = 62)

Structures **c1** and **c9** (Figures S7 and 8) resemble hydrogenated methyl isocyanate (CH_3_NCO), and **c10** (Figure S7) is identical to NH_2_ CH_2_CO, a glycine precursor. In this case, we do not have molecular geometries based on the molecular skeleton of methanol, that is, with the CN group interacting with the hydroxyl or methyl group. Structures **c3** and **c5** resemble the CHOH group associated with H_2_CN, through hydrogen atoms migrating from the methyl group to the CN group. Since there was no distinction between deuterium and hydrogen atoms, the optimized stoichiometry was the same as in the case presented earlier, CH_3_CN + OD.

#### 4.3.4 CD_3_OD + HCN (*m*/*z* = 63)

The methanol molecule associated with cyanic acid (HCN) can be formed from the fragmentation of the ACN molecule and further hydrogenation of the CN group. This stoichiometry is equivalent to two molecules identified in the ISM:^43^ acetamide (CH_3_ CONH_2_) and the n-methyl formamide (CH_3_NHCHO). Structures **d3**, **d5**, and **d10** (Figures S8 and 8) are similar to acetamide, with hydrogen migrating from the methyl group to the nitrogen or oxygen atoms. Structures **d7**, **d8**, and **d9** are close to n-methyl formamide, with one hydrogen migrating from methyl to the oxygen atom. Some structures such as **d1** resemble the geometry of the glycine precursor NH_2_ CH_2_CO, with the same N–C–C–O chain. The molecular geometry depends on the number of hydrogen atoms, but in this case, the basic molecular unity is preserved.

## 5 Conclusions

The spectral signatures observed by the TOF and TPD techniques revealed the complexity of the structures and the interaction between the reacting molecules in the condensed phase, which should be taken into account when chemical reactions are studied under similar conditions. The TPD spectra aided in the evaluation of the temperature of thermal desorption. The TPD profile of the codeposited film showed closer sublimation of the molecules, suggesting better mixing of the compounds.

The results showed that intermolecular reactions and the formation of larger molecules take place in mixed films of MeOD and ACN. The TOF technique provided information about the ionic production following electron bombardment. Several ionic species were detected, produced by the breakup of the interacting molecules. Those species helped us to point out through which channels the intramolecular and intermolecular interactions of methanol and acetonitrile proceeded.

The attachment and exchange of H and D atoms was the most relevant process in species production, although fragment radicals also formed several species. In pure methanol films, the clusterization is fully active, but on the other hand, only the ACN dimer was formed in the pure acetonitrile spectrum. Cluster formation was more pronounced from the codeposited mixtures, indicating that this molecular film structure allowed a higher degree of intermolecular interaction by the electron-driven desorption process.

Ionic species not produced in pure films were produced in both codeposited and bilayer films after annealing (diffusion and scrambling of molecules), as witnessed by the formation of larger molecules. Therefore, the annealing process was essential to enhance the interaction between the molecules, greatly favoring H/D exchanges, radical attachments, and cluster formation. Annealing led to a larger variety of species and was responsible for the production of most of the aggregates in the bilayer mixture, since almost none of these species were formed before the procedure was applied.

Moreover, the production of mixed clusters provided information about the formation routes of complex organic molecules, which have already been detected in many astronomical environments. The optimized structures presented hold similarities to many species identified by radio astronomy, with an emphasis on the amino acid glycine, which is still sought in the ISM. It is clear that the acetonitrile and methanol mixtures in the condensed phase provide a rich environment that helps to explain the variety of organic molecules detected in different media. Furthermore, the observed spectral signatures from the mixed molecular films may help to model the intermolecular and intramolecular interactions and the energetic processing of ices composed of different molecules under the impact of particles and to better understand their influence on ionic production and desorption under more realistic conditions.

## Supporting Information Available

Injection profile of the codeposited mixture; TPD spectra of the pure films; penetration depth distribution simulated with the aid of the CASINO software of 2.3 keV electrons with a discussion of the results; all calculated GM structures for the cluster species discussed in the main text; raw mass-to-charge spectra of pure MeOD, pure acetonitrile, and MeOD/ACN in codeposited and bilayer films without and with the annealing process; ratios between the yields of the hydrogenated and deuterated forms of the methanol clusters for different films; and possible molecular structures of different mass-to-charge ratios (PDF)
