# Supplementary material for: Ionic Fragments and Clusters Produced by Electron Impact of Acetonitrile and Methanol Mixed Molecular Films
Source: J Phys Chem A. 2025 Apr 2;129(15):3389–400. doi: 10.1021/acs.jpca.4c08285 (PMC12010316; doi:10.1021/acs.jpca.4c08285)
Supplement: Supplementary file 1 — jp4c08285_si_001.pdf [file jp4c08285_si_001.pdf]

Supporting Information: Ionic Fragments and Clusters Produced by Electron Impact of Acetonitrile and Methanol Mixed Molecular Films

Wania Wolff,*,† Andre M.R. Giraldi,†,§ Jorge H. C. Basilio,† Fabio de A. Ribeiro,‡ Alvaro Nunes Oliveira,† and Ricardo R. Oliveira¶

†*Physics Institute, Federal University of Rio de Janeiro, Rio de Janeiro, RJ 21941-909, Brazil.*

‡*Federal Institute of Rio de Janeiro, Nilópolis, RJ 26530-060, Brazil.*

¶*Chemistry Institute, Federal University of Rio de Janeiro, Rio de Janeiro, RJ 21941-909, Brazil.*

§*Max-Planck-Institut für Kernphysik, Heidelberg, DE-69117, Germany*

E-mail: wania@if.ufrj.br

Contents

1 Injection spectra **S-3**

2 TDP of pure ACN and MeOD **S-4**

3 Electron trajectories simulations within the layered ices **S-4**

4 All structures **S-7**

5 Mass spectra of deuterated methanol - acetonitrile mixture **S-9**

6 Yields of the hydrogenated and deuterated form of methanol clusters **S-13**

7 Assignment of each mass-to-charge ratio to a fragment. **S-14**

References **S-17**

1 Injection spectra

We determine the number of monolayers (L=1×10^{-6})Torr seconds deposited during the codeposition and bilayer processes by integrating in time the spectra of ACN^{+} and $MeOD^{+}$ (see figure S1 and multiplied by the ratio of the absolute total and single ionized cross sections of molecules measured in the gas phase available in the literature.[S1–S4] The pressure was measured by QMS-RGA at 70 eV in steps of 2 seconds and the data was recorded using the labview software.

$$nL(10^{-6}Torrsec) = \left(\int_{t1}^{t2} P_{ressure}(t)dt\right) \otimes \frac{\sigma_{Total}}{\sigma_{Mol^{+}}} \otimes 0.75 \qquad (1)$$

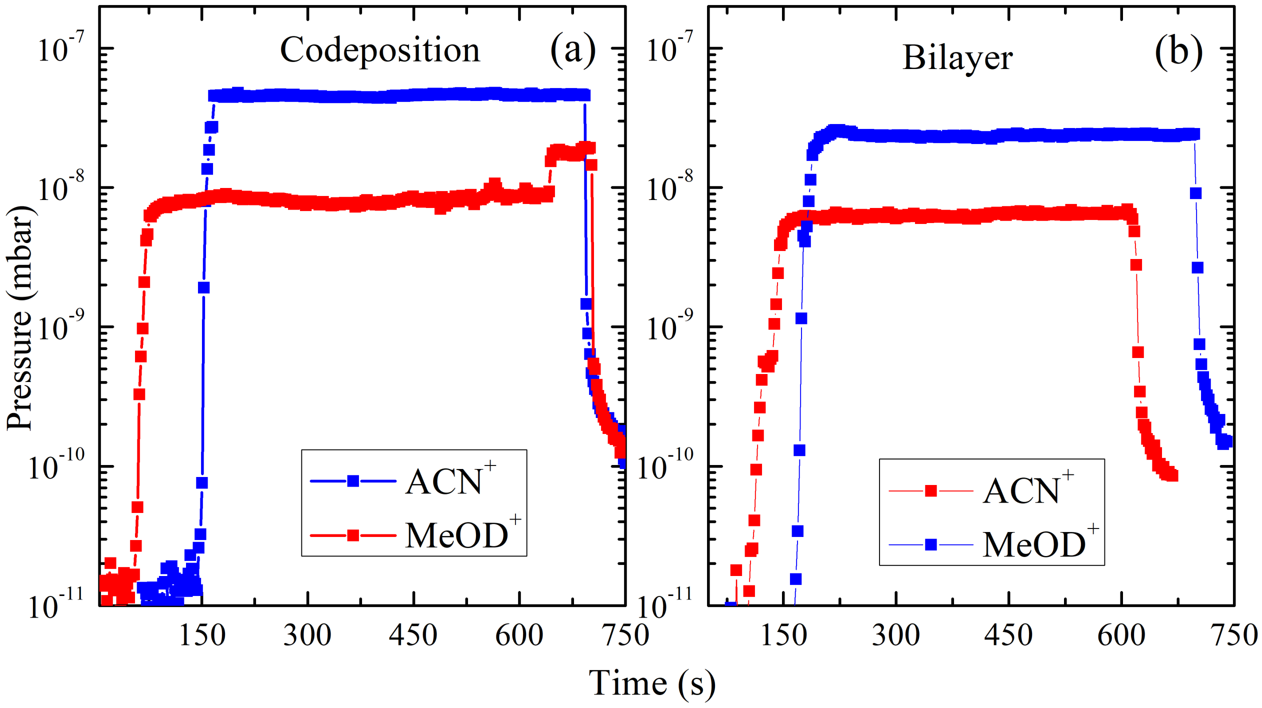

Figure S1: Injection spectra given in pressure (mbar) versus time (seconds) of ionized ACN and MeOD indicated by red and blue solid lines respectively in the (a) codeposition and (b) bilayer process.

2 TDP of pure ACN and MeOD

We show the TPD spectra (figure S2 (a) and (b) of pure ACN and MeOD indicating the temperature of the sublimation peak by the dashed lines. The pressure was measured by QMS-RGA at 70 eV, and the temperature was measured by a type K thermocouple monitored by the Eurothrem controller. Data acquisition and recording was performed using labview software.

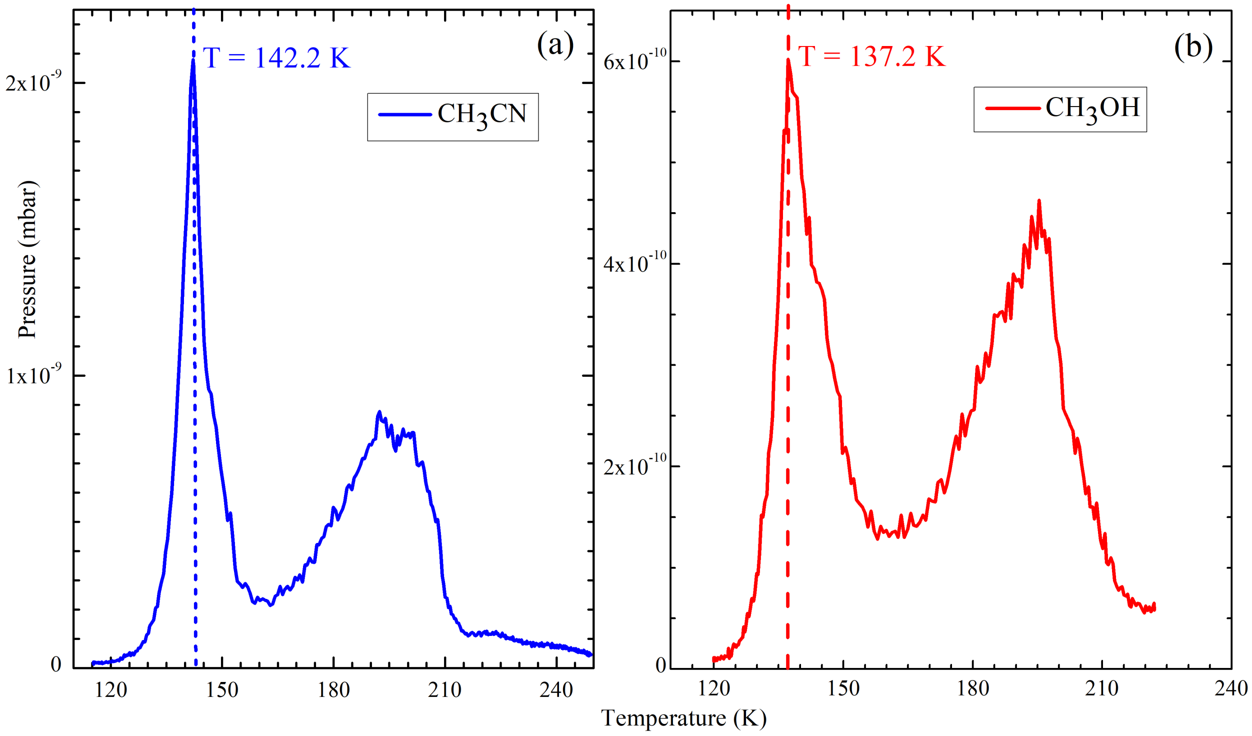

Figure S2: TPD spectra of (a) pure acetonitrile (b) pure methanol

3 Electron trajectories simulations within the layered ices

We used the CASINO code to simulate the penetration depth of electrons within the ices studied in this work. The maximum distribution depth, its mean value, and the maximum of the distribution are quite similar for pure and layered ices, so only information about the sequentially deposited ices (bilayer regime) shall be given here.

As discussed in the experimental details of the main article, ice thickness was determined as

monolayer (ML) equivalent resulting from the exposure of the cold substrate to sample vapor (as from the dosing curves presented in Figure S1). The resulting exposures for the bilayer deposition yield a thickness of about 23 ML for acetonitrile (ACN, CH_3CN) and 9.5 ML for deuterated methanol (MeOD, CD_3OD). In order to derive the thickness of each layer in the nanometer scale as input for the simulations, we made a rough estimation by assuming that each monolayer has dimensions very close to the dimensions of the unit cell of each molecular crystal.

The crystal structure of methanol in the nanometer scale has been examined in detail by He et al.[S5] using reflection high-energy electron diffraction (RHEED), where the lattice constants $a = 4.71$ Å, $b = 4.93$ Å, $c = 9.13$ Å, have been determined at 145 K. Even though this temperature is considerably higher than the one used here for the ice growth, the authors argued that the cell parameters derived are in good agreement with previous measurements made by Kirchner et al.[S6] for bulk methanol at 122 K. Thus, by considering the stacking direction of molecular layers vertically along the c direction, we assume a thickness of $\approx 0,913$ nm for the MeOD monolayer at 120 K and a total thickness of ≈ 8.7 nm for the MeOD layer directly deposited on the cold stainless steel substrate. Likewise, the lattice constants of ACN single crystal have been derived for the α-phase by[S6] as $a = 4.102$ Å, $b = 8.244$ Å, $c = 7.970$ Å, and the c parameter was also used as an estimation of the length of one single ACN layer. Thus, from the exposure used for growing ACN on top of MeOD at 120 K, the thickness of the ACN layer is estimated to be ≈ 18.1 nm thick and the total thickness of the molecular film can be estimated as ≈ 26.8 nm as an upper limit. These values were used as reference for the electron trajectories simulations under layered ice structure.

The bulk density of ACN was taken for the low-temperature crystalline ice as $1.058\ \mathrm{g \cdot cm^{-3}}$.[S7] Specific measurements for the density of crystalline perdeuterated methanol (MeOD) at 120 K were not found, so the bulk density of methanol (CH_3OH, MeOH)[S8] was used multiplied by the ratio of molar masses between MeOD and MeOH, resulting in $1.152\ \mathrm{g \cdot cm^{-3}}$ (supposing a similar packing and number density per unit cell). The MeOD layer is supported on top of the substrate, considered here as high-purity stainless steel with a density of $7.7\ \mathrm{g \cdot cm^{-3}}$. Finally, the incident electron beam impinges onto the sample surface with an angle of 60° with respect to the surface

normal. The incident electron penetration depth distribution as simulated is shown in Figure S3.

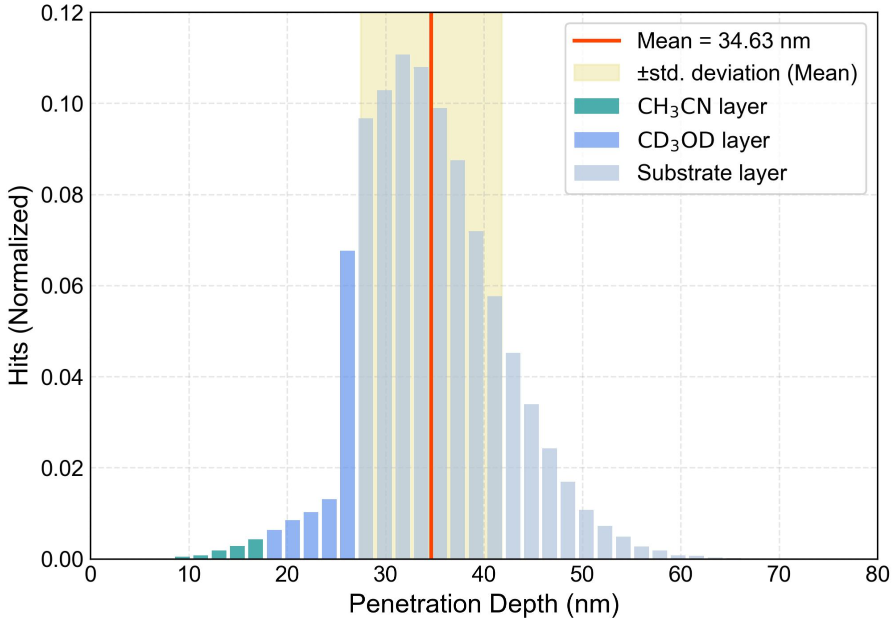

Figure S3: Penetration depth distribution simulation for electrons with 2.3 keV impact energy impinging on a solid CH_3CN layer on top of solid CD_3OD (18.1 nm and 8.7 nm thick, respectively). The layered sample is supported on a stainless-steel substrate. The length of each layer is represented by a different color bar scheme. The mean penetration depth is marked by a red vertical line and the pale-yellow area encloses the standard deviation of the mean penetration depth (34.63 $\pm$ 7.18 nm).

The bars of the distribution shown in Figure S3 are rendered in three different colors to illustrate the depth of each simulated layer. As seen from the distribution in Figure S3, electrons can penetrate deeper than the thickness of the molecular film. The maximum penetration depth of the electron beam extends up to about 80 nm, and its maximum at 31.74 nm is located close to the interface between the MeOD layer and the substrate. The red vertical line indicates the mean penetration depth of the electrons, and the region highlighted behind is the standard deviation of the mean penetration depth (66. 25 $\pm$ 25.01 nm).

Even though some projectile particles may be eventually scattered and stopped within the film, the majority of the electrons transverse the film and reach the substrate of the sample holder.

4 All structures

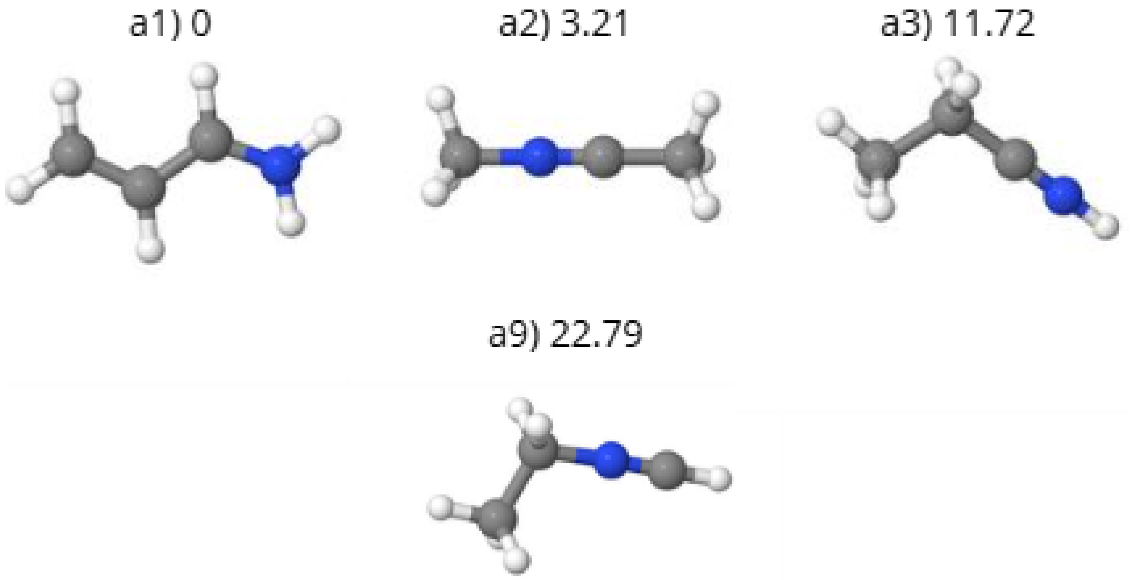

Figure S4: Calculated GM structures for $CH_3CN + CH_3$. The relative energies are in kcal mol^{-1}.

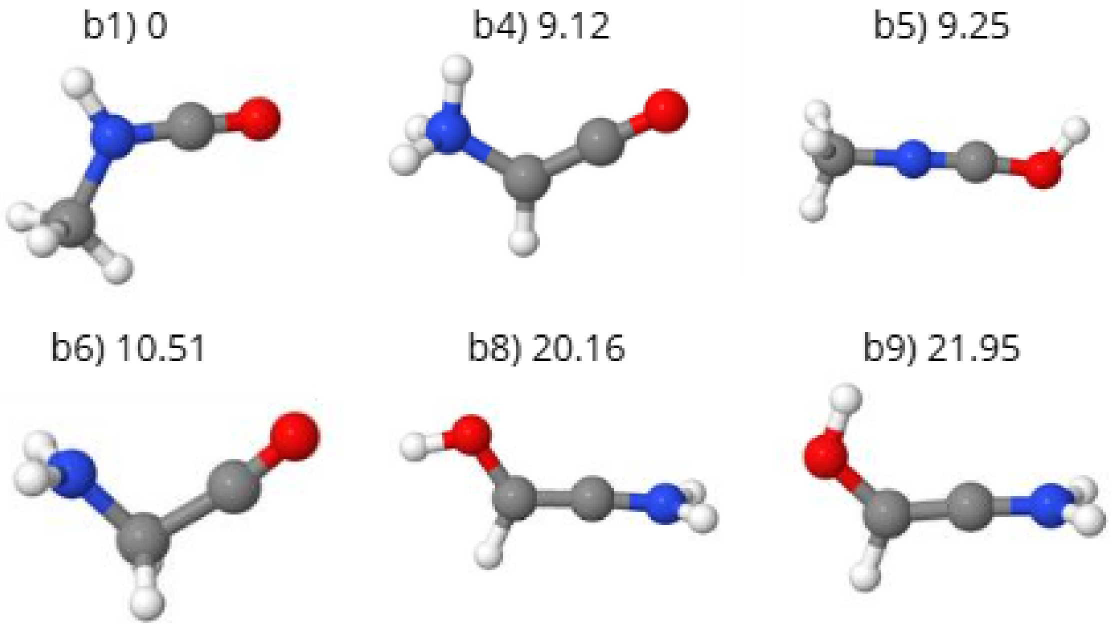

Figure S5: Calculated GM structures of $CH_3CN + OH$.The relative energies are in kcal mol^{-1}.

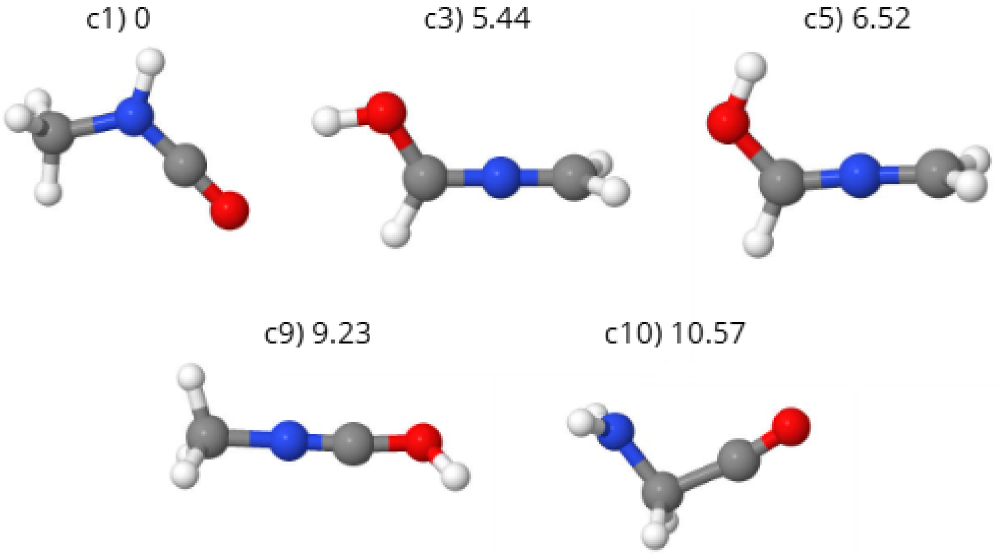

Figure S6: Calculated GM structures of CH_3OH + CN.The relative energies are in kcal mol^{-1}.

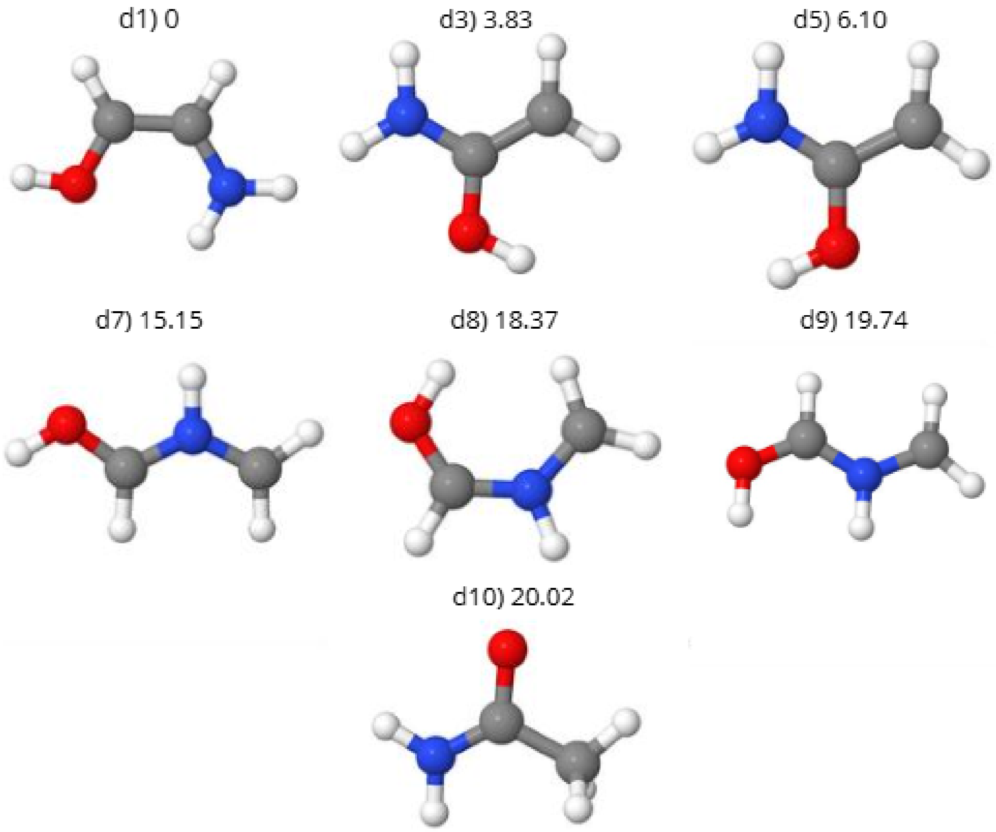

Figure S7: Calculated GM structures of CH_3OH + HCN.The relative energies are in kcal mol^{-1}.

5 Mass spectra of deuterated methanol - acetonitrile mixture

The ion yields were derived from the raw time-of-flight spectra under the incidence of 2300 eV electrons. Examples of typical spectra collected from the spectrometer are shown in Figures S8 - S13. The yield of each mass is derived by computing the area using Gaussian fits of the time of flight signal with respect to the background level.

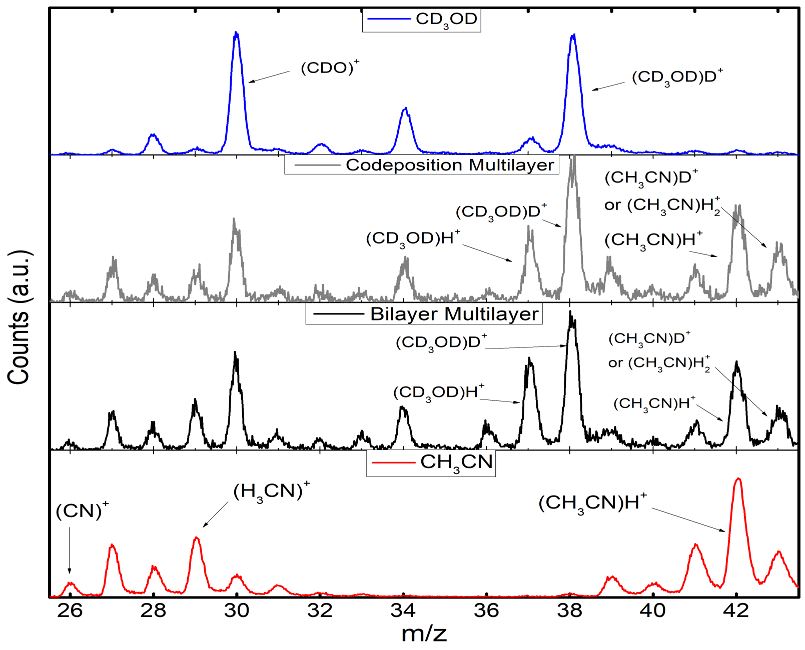

Figure S8: Mass/charge spectra, from top to bottom, of pure MeOD, codeposited mixture, bilayer mixture and pure ACN in the range of m/z 26 to 43 covering only the fragmentation pattern.

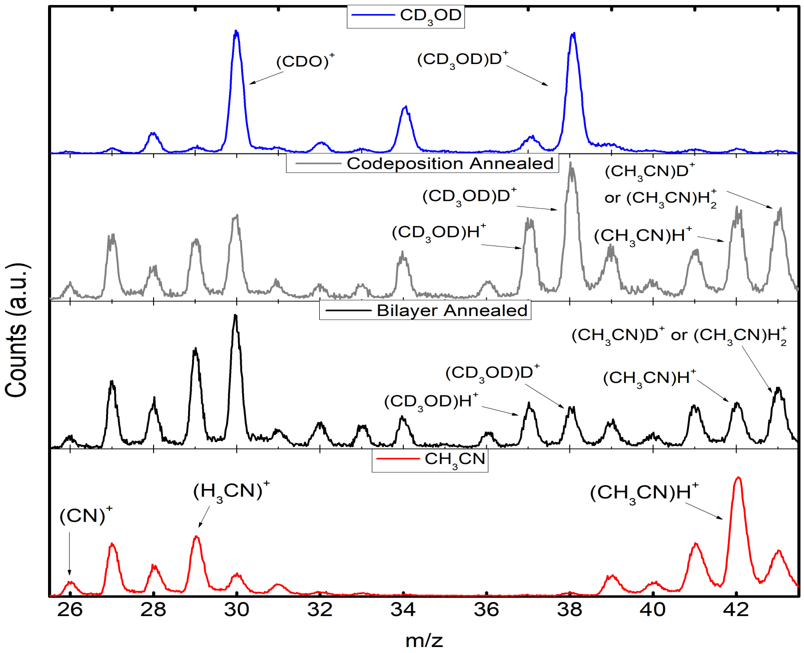

Figure S9: Mass/charge spectra, from top to bottom, of pure MeOD, codeposited mixture after annealing, bilayer mixture after annealing and pure ACN in the range of m/z 26 to 43, covering the same range as Figure ref {fig1

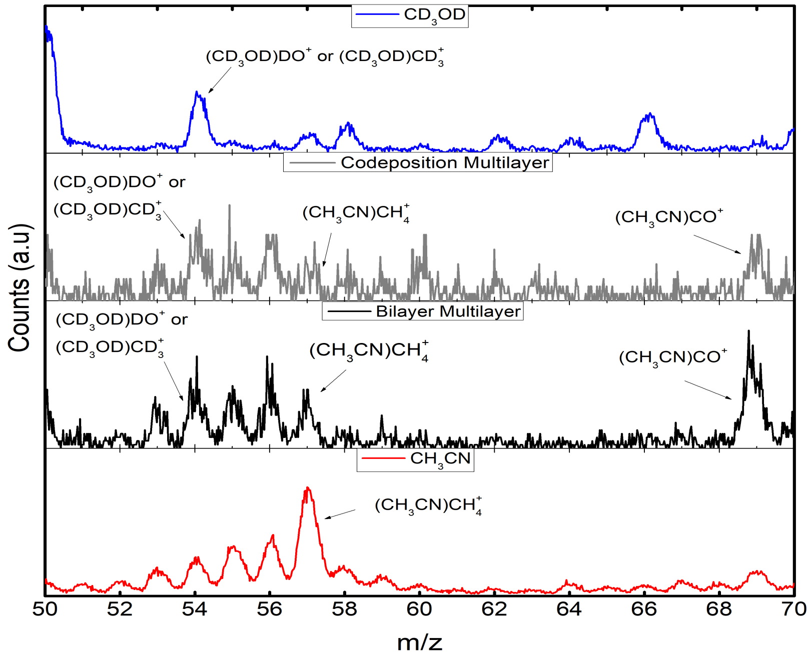

Figure S10: Mass/charge spectra, from top to bottom, of pure MeOD, codeposited mixture, bilayer mixture and pure ACN in the range of m/z 50 to 70.

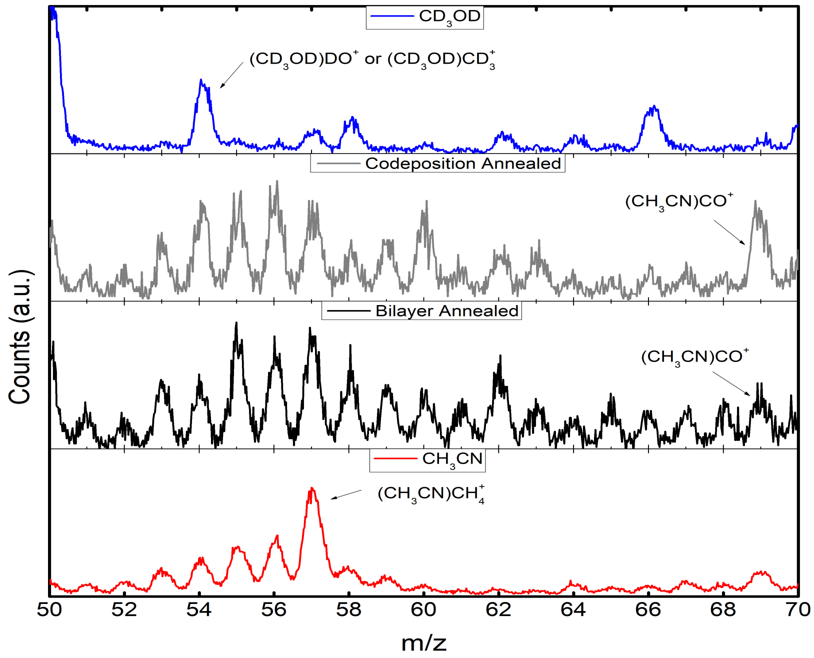

Figure S11: Mass/charge spectra, from top to bottom, of pure MeOD, codeposited mixture after annealing, bilayer mixture after annealing and pure ACN in the range of m/z 50 to 70.

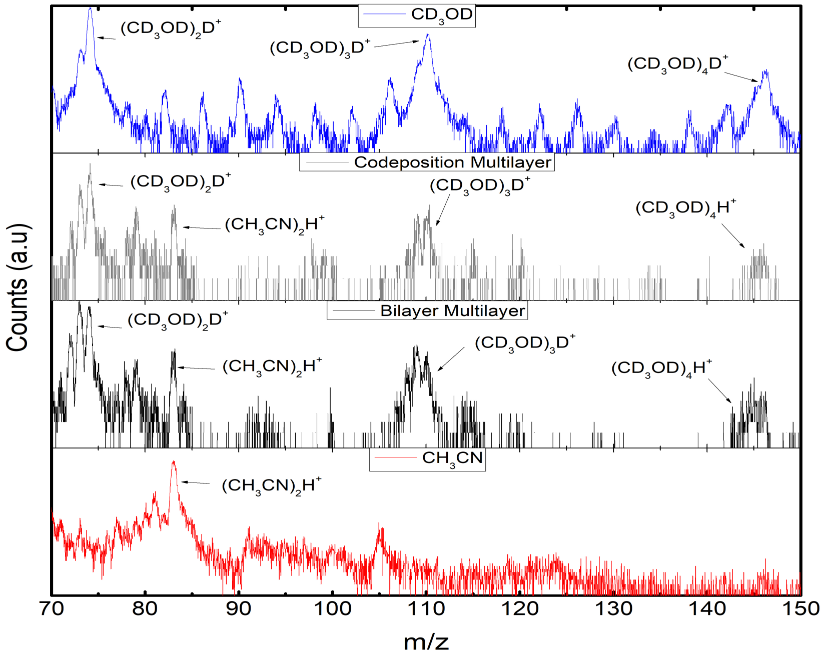

Figure S12: Mass/charge spectra, from top to bottom, of pure MeOD, codeposited mixture, bilayer mixture and pure ACN in the range of m/z 70 to 150.

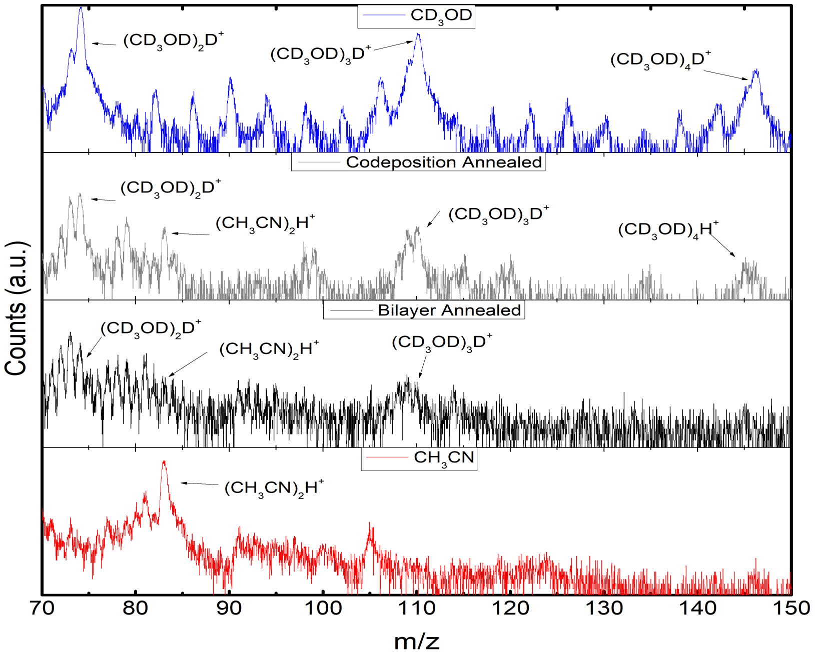

Figure S13: Mass/charge spectra, from top to bottom, of pure MeOD, codeposited mixture after annealing, bilayer mixture after annealing and pure ACN in the range of m/z 70 to 150.

6 Yields of the hydrogenated and deuterated form of methanol clusters

We present the ratios between the yields of the hydrogenated and deuterated forms of methanol clusters for the pure and codeposited and bilayer film. The ratios were obtained considering different amounts of methanol units (n) using the formula: $(CD_3OD)_nH^+/(CD_3OD)_nD^+$.

Table S1: Ratios between the yields of the hydrogenated and deuterated form of methanol clusters for each of the analyzed films.

Number of units (n)	**Pure Methanol**	**Codeposited Multilayer**	**Codeposited Annealed**	**Bilayer Multilayer**	**Bilayer Annealed**
1	0.14	0.43	0.60	0.65	1.02
2	0.21	0.50	0.73	1.05	1.33
3	0.20	0.56	0.68	1.44	1.90
4	0.30	1.62	1.02	1.85	-

7 Assignment of each mass-to-charge ratio to a fragment.

In the following tables, we suggest the assignment of the mass-charge ratios to possible molecular structures.

Table S2: Assignment of mass-to-charge ratio from m/z = 50 to m/z = 69.

m/z	Assignments	m/z	Assignments
50	$(CD_3OD)CD$	60	$(CD_3OD)C_2$
51	$(CD_3OD)CH_3$	61	$(CH_3CN)D_2O$
52	$(CH_2CN)C$	62	$(CD_3OD)CN$
53	$(CH_3CN)C$	63	$(CD_3OD)HCN$
54	$(CH_3CN)CH$ or $(CD_3OD)CD_3$ or $(CD_3OD)OD$	64	$(CD_3OD)H_2CN$
55	$(CH_3CN)CH_2$	65	$(CD_3OD)H_3CN$
56	$(CH_3CN)CH_3$ or $(CD_3OD)D_2O$	66	$(CD_3OD)H_3CNH$
57	$(CH_3CN)CH_4$	67	$(CD_3OD)H_3CNH_2$
58	$(CD_3OD)D_3O$	68	$(CD_3OD)H_3CNH_3$
59	$(CH_3CN)CD_3$ or $(CD_3OD)OD$	69	$(CH_3CN)CO$

Table S3: Assignment of mass-to-charge ratio from m/z = 72 to m/z = 86 and from m/z = 91 to m/z = 95.

**m/z**	**Assignments**	**m/z**	**Assignments**
72	$(CD_3OD)_2$	82	$(CH_3CN)_2$
73	$(CD_3OD)_2H$	83	$(CH_3CN)_2H$
74	$(CD_3OD)_2D$	84	$(CH_3CN)_2H_2$ or $(CH_3CN)_2D$
75	$(CD_3OD)_2DH$	85	$(CH_3CN)_2DH$
76	$(CD_3OD)_2D_2$	86	$(CH_3CN)_2D_2$
77	$(CH_3CN)(CD_3OD)$	91	$(CD_3OD)_2(COH)$
78	$(CH_3CN)(CD_3OD)H$	92	$(CD_3OD)_2(COD)$
79	$(CH_3CN)(CD_3OD)D$	93	$(CD_3OD)_2(CODH)$
80	$(CH_3CN)CHCN$	94	$(CD_3OD)_2(CDOD)$
81	$(CH_3CN)CH_2CN$	95	$(CD_3OD)_2(CDODH)$

Table S4: Assignment of high mass-to-charge ratio of selected fragments.

**m/z**	**Assignments**	**m/z**	**Assignments**
97	$(CD_3OD)_2C_2H$	115	$(CH_3CN)(CD_3OD)_2D$
98	$(CD_3OD)_2C_2D$	118	$(CH_3CN)_2(CD_3OD)$
99	$(CD_3OD)_2C_2DH$	119	$(CH_3CN)_2(CD_3OD)H$
108	$(CD_3OD)_3$	120	$(CH_3CN)_2(CD_3OD)D$
109	$(CD_3OD)_3H$	144	$(CD_3OD)_3$
110	$(CD_3OD)_3D$	145	$(CD_3OD)_3H$
113	$(CH_3CN)(CD_3OD)_2$	146	$(CD_3OD)_3D$
114	$(CH_3CN)(CD_3OD)_2H$	-	-

References

(S1) Parkes, M. A.; Douglas, K. M.; Price, S. D. Ionization of acetonitrile. *International Journal of Mass Spectrometry* **2019**, *438*, 97–106.

(S2) Zhou, W.; Wilkinson, L.; Lee, J. W. L.; Heathcote, D.; Vallance, C. Total electron ionization cross-sections for molecules of astrochemical interest. *Molecular Physics* **2019**, *117*, 3066–3075.

(S3) Rejoub, R.; Morton, C. D.; Lindsay, B. G.; Stebbings, R. F. Electron-impact ionization of the simple alcohols. *The Journal of Chemical Physics* **2003**, *118*, 1756–1760.

(S4) Nixon, K.; Pires, W.; Neves, R.; Duque, H.; Jones, D.; Brunger, M.; Lopes, M. Electron impact ionisation and fragmentation of methanol and ethanol. *International Journal of Mass Spectrometry* **2016**, *404*, 48–59.

(S5) He, X.; Wu, C.; Yang, D. Communication: No guidance needed: Ordered structures and transformations of thin methanol ice on hydrophobic surfaces. *The Journal of Chemical Physics* **2016**, *145*, 171102.

(S6) Kirchner, M. T.; Das, D.; Boese, R. Cocrystallization with Acetylene: Molecular Complex with Methanol. *Crystal Growth & Design* **2008**, *8*, 763–765.

(S7) Hudson, R. L. Preparation, identification, and low-temperature infrared spectra of two elusive crystalline nitrile ices. *Icarus* **2020**, *338*, 113548.

(S8) Hudson, R. L.; Gerakines, P. A.; Yarnall, Y. Y. Infrared Spectroscopic and Physical Properties of Methanol Ices—Reconciling the Conflicting Published Band Strengths of an Important Interstellar Solid. *The Astrophysical Journal* **2024**, *970*, 108.
